# Effects of probiotic supplementation on diabetic kidney disease: a systematic review and meta-analysis of randomized controlled trials

**DOI:** 10.3389/fmicb.2026.1760954

**Published:** 2026-05-20

**Authors:** Zhi-Qing Huang, Huai-Yu Li, Yan-Ting Li, Ruo-Xin Zhang, En Zhang, Bao-Lin Su

**Affiliations:** 1 The First Clinical College, Guangzhou University of Chinese Medicine, Guangzhou, China; 2 Guangdong Clinical Research Academy of Chinese Medicine, Guangzhou, China; 3 Department of Nephrology, The First Affiliated Hospital of Guangzhou University of Chinese Medicine, Guangzhou, China

**Keywords:** complementary therapy, diabetic kidney disease, meta-analysis, probiotics, systematic review

## Abstract

**Background:**

Diabetic kidney disease (DKD) is one of the leading causes of end-stage kidney disease worldwide, and microbiota-based interventions are considered potential preventive and therapeutic strategies to slow its progression. This study evaluated the efficacy of probiotic supplementation in patients with DKD through a systematic review and meta-analysis.

**Methods:**

We searched seven databases, including PubMed, Embase, the Cochrane Library (CENTRAL), CNKI, Wanfang, VIP and SinoMed, to identify all relevant studies published from database inception to July 2025. Eligible studies were randomized controlled trials assessing probiotic interventions in DKD patients. Study quality was evaluated using the Cochrane Risk of Bias tool, and meta-analyses were performed using R.

**Results:**

A total of 11 studies involving 726 participants were included. Probiotic supplementation significantly improved serum creatinine (MD − 0.17, −0.22 to −0.12, *p* < 0.0001) and estimated glomerular filtration rate (MD 9.62, 2.74 to 16.51, *p* < 0.0001). No significant effects were observed for blood urea nitrogen, urinary albumin-to-creatinine ratio, 24-h urinary protein or cystatin C. Probiotics also showed notable benefits in glycemic and lipid parameters, including reductions in fasting plasma glucose (MD − 18.52, −27.08 to −9.97, *p* < 0.0001), glycated hemoglobin (MD − 0.18, −0.31 to −0.05, *p* = 0.009), and homeostasis model assessment of insulin resistance (MD − 1.22, −2.01 to −0.43, *p* = 0.002), as well as an increase in high-density lipoprotein cholesterol (MD 5.45, 1.08 to 9.83, *p* = 0.01). In addition, probiotic supplementation led to significant reductions in inflammatory and oxidative stress markers, including high-sensitivity C-reactive protein (MD − 1.53, −2.38 to −0.69, *p* = 0.0004) and malondialdehyde (MD − 0.81, −0.97 to −0.65, *p* < 0.0001), and a significant increase in total antioxidant capacity (MD 62.71, 41.80 to 83.62, *p* < 0.0001).

**Conclusion:**

Probiotic supplementation may improve metabolic disturbances, renal function, and systemic inflammation/oxidative stress in DKD patients. However, its clinical efficacy and long-term effects require further validation in large-scale studies.

## 1 Introduction

Diabetic kidney disease (DKD) is one of the most common microvascular complications of diabetes and represents a major attributable cause of chronic kidney disease (CKD). It is clinically characterized by the presence of persistent proteinuria accompanied by a progressive reduction in estimated glomerular filtration rate (eGFR) (Tuttle et al., 2022). According to the classical pathophysiological model of DKD, long-term hyperglycemia triggers intrarenal oxidative stress, activation of inflammatory cascades, and metabolic derangements, subsequently leading to podocyte injury, glomerulosclerosis, and tubulointerstitial fibrosis, and eventually resulting in irreversible structural damage to the kidney (DeFronzo et al., 2021). Based on predictive modeling from the Global Burden of Disease 2021 study, by 2050, more than 1.31 billion people worldwide are projected to be living with diabetes, further escalating the global burden of chronic kidney failure and cardiovascular events (GBD 2021 Diabetes Collaborators, 2023). Although multiple evidence-based therapeutic strategies have demonstrated substantial efficacy in slowing DKD progression in recent years, a proportion of patients continues to deteriorate rapidly despite optimal management, highlighting the inherent limitations of conventional strategies that primarily target renal structural pathology (Alicic et al., 2017; Lytvyn et al., 2020). Consequently, the pursuit of novel therapeutic targets has become a major focus of both basic and clinical research in DKD.

The gut microbiota is a highly dynamic and interdependent microbial ecosystem composed of more than 100 trillion microorganisms (Qin et al., 2010). Under physiological conditions, it maintains a symbiotic relationship with the host: while the host provides a supportive habitat and essential nutrients, the microbial community contributes to host homeostasis by preventing pathogen colonization, participating in nutrient metabolism, and modulating mucosal immunity. By contrast, disturbances in the composition and architecture of the gut microbiota are increasingly linked to a higher risk of chronic diseases (Lynch and Pedersen, 2016), including CKD (Jin et al., 2026). As renal function declines, retention of uremic solutes, dietary alteration, and drug exposure progressively reshape the gut microbial community and its metabolic output (Koppe et al., 2018; Mertowska et al., 2021; Deng et al., 2022). Available evidence indicates that CKD-associated dysbiosis is not defined by isolated shifts in individual taxa, but by a broader ecological disruption characterized by loss of beneficial commensals, expansion of opportunistic pathogens, and disordered microbial metabolism (Chung et al., 2019; Li et al., 2024). Patients with CKD typically show increased abundances of *Proteobacteria/Enterobacteriaceae*, *Enterococcaceae*, and *Streptococcus*, together with depletion of *Firmicutes*, *Prevotellaceae*/*Prevotella*, and several short-chain fatty acid (SCFA)–producing bacteria (Bloom et al., 2025). In end-stage renal disease, this deleterious microbial profile becomes more pronounced, with further reductions in butyrate-producing taxa, including *Roseburia*, *Faecalibacterium prausnitzii*, and *Eubacterium rectale*, alongside enrichment of *Eggerthella lenta*, *Fusobacterium*, and bacteria involved in aromatic amino acid catabolism (Wong et al., 2014; Wang et al., 2020; Laiola et al., 2025). Gut microbial disturbances also display a degree of subtype specificity across CKD. In IgA nephropathy, patients commonly show enrichment of *Firmicutes*, particularly *Ruminococcaceae* and *Lachnospiraceae*, together with depletion of beneficial genera such as *Clostridium*, *Lactobacillus*, and *Bifidobacterium* (Gesualdo et al., 2021; Tan et al., 2022). In membranous nephropathy, increases in *Proteobacteria*, *Actinobacteria*, *Escherichia-Shigella*, and *Subdoligranulum* have been reported, whereas *Bacteroidota*, *Bacteroides*, *Prevotella*, and *Megamonas* are reduced (Shang et al., 2022). Among CKD subtypes, DKD, the most common and clinically representative form, has been studied most extensively. Available evidence indicates that DKD is generally associated with a lower *Firmicutes-to-Bacteroidetes* ratio, along with enrichment of *Clostridium*, *Shigella*, *Bilophila*, *Acidaminococcus*, *Escherichia*, *Megasphaera*, *Veillonella*, and *Escherichia-Shigella*. By contrast, *Lachnospira*, *Intestinibacter*, and several butyrate-producing taxa, including *Eubacterium* and *Roseburia intestinalis*, are consistently depleted (Jin et al., 2026). Mechanistically, disruption of gut microbial metabolism is a central pathogenic link between intestinal dysbiosis and kidney injury in CKD. As renal function declines, microbial metabolism shifts from saccharolytic fermentation toward proteolytic fermentation and aromatic amino acid catabolism, with a parallel reduction in protective metabolites and accumulation of uremic toxins and their precursors (Bloom et al., 2025). Gut-derived metabolic disturbances and CKD therefore form a self-perpetuating, bidirectional network. Reduced kidney function promotes urea retention, impairs intestinal barrier integrity, and alters the luminal milieu, thereby driving the microbiota toward a toxin-producing phenotype. In turn, depletion of protective metabolites, including SCFAs and indole-3-aldehyde, together with accumulation of injurious metabolites such as indoxyl sulfate (IS), *p*-cresyl sulfate (*p*CS), indole-3-acetic acid (IAA), and trimethylamine *N*-oxide (TMAO), further amplifies oxidative stress, inflammation, and fibrosis through aryl hydrocarbon receptor (AHR), nuclear factor kappa B (NF-κB), Kelch-like ECH-associated protein 1 (Keap1)/nuclear factor erythroid 2–related factor 2 (Nrf2), transforming growth factor-beta (TGF-*β*)/Smad, and NOD-like receptor family pyrin domain containing 3 (NLRP3) signaling pathways. These processes collectively accelerate disease progression. Accordingly, precision management of DKD should extend beyond the kidney itself. Microbiota-targeted interventions may offer a promising strategy to delay DKD progression by restoring gut microbial homeostasis and rebalancing microbially derived metabolites.

Probiotics are live microorganisms that colonize the host and contribute to health maintenance. As a microbiota-targeted intervention, probiotics have shown promise in CKD. In patients receiving peritoneal dialysis, probiotic supplementation may improve inflammatory status, nutritional indices, and quality of life, and might reduce the risk of peritoneal dialysis-associated peritonitis (Stepanova, 2024). In DKD, interest has similarly grown in their potential effects on kidney function, glucose and lipid metabolism, and inflammatory markers. These benefits may arise through remodeling of the gut microbiota, modulation of microbial metabolites, restoration of intestinal barrier integrity, and attenuation of inflammation, thereby contributing to renoprotection (Huang and Chen, 2024). However, clinical studies in DKD remain limited and heterogeneous, and a systematic synthesis of the available evidence is still lacking. Here, we conducted a systematic review and meta-analysis to comprehensively evaluate the therapeutic effects of probiotic supplementation in DKD, thereby providing stronger evidence to guide its potential clinical use.

## 2 Methods

This study was conducted in accordance with the Preferred Reporting Items for Systematic Reviews and Meta-Analyses (PRISMA) guidelines (Page et al., 2021). The study protocol was prospectively registered in the International Prospective Register of Systematic Reviews (PROSPERO; https://www.crd.york.ac.uk/PROSPERO/, registration number CRD420251180316).

### 2.1 Search strategy

Two reviewers (Y-TL and R-XZ) independently performed a comprehensive literature search in PubMed, Embase, the Cochrane Library (CENTRAL), the China National Knowledge Infrastructure (CNKI), Wanfang Data, the VIP Database, and the Chinese Biomedical Literature Database (SinoMed) to identify studies evaluating probiotic therapy for DKD. All databases were searched from inception to July 2025. Both Medical Subject Headings terms and free-text keywords were used, and the complete search strategies are detailed in Supplementary Table S1.

### 2.2 Inclusion and exclusion criteria

Only randomized controlled trials (RCTs) evaluating the therapeutic effects of probiotics in patients with DKD were included in this study. In the control group, participants received standard therapy alone or standard therapy plus placebo, whereas the intervention group received probiotic supplementation in addition to the same standard treatment. Studies were eligible if they reported at least one of the following outcomes: (1) renal function markers, including serum creatinine (Scr), eGFR, blood urea nitrogen (BUN), urinary albumin-to-creatinine ratio (UACR), 24-h urinary protein (24 h UP), and cystatin C (Cys-C); (2) glycemic metabolism markers, including fasting plasma glucose (FPG), glycated hemoglobin (HbA1c), homeostasis model assessment of insulin resistance (HOMA-IR), and 2-h postprandial glucose (2 h-PG); (3) lipid metabolism markers, including triglycerides (TG), total cholesterol (TC), high-density lipoprotein cholesterol (HDL-C), and low-density lipoprotein cholesterol (LDL-C); and (4) inflammation and oxidative stress markers, including high-sensitivity C-reactive protein (hs-CRP), malondialdehyde (MDA), total antioxidant capacity (TAC), and nitric oxide (NO). The safety of probiotic therapy in patients with DKD was also assessed. For multi-arm trials, if more than one intervention arm met the inclusion criteria, each eligible arm was included as an independent comparison in accordance with the Cochrane Handbook; when multiple arms shared a common control group, the control sample size was proportionally divided to avoid unit-of-analysis errors arising from double counting. Studies were excluded if they were non-RCTs, failed to report relevant outcomes or interventions, or included populations that did not meet the predefined eligibility criteria.

### 2.3 Data extraction

Study selection and data extraction were performed independently by two reviewers (Z-QH and H-YL). Duplicate records were first removed using EndNote X9, after which titles, abstracts, and full texts were screened sequentially according to the predefined inclusion and exclusion criteria. Any disagreements were resolved through discussion or adjudication by a third reviewer (EZ or B-LS). Data were extracted into a standardized Excel spreadsheet, including publication year, sample size, baseline characteristics of participants, probiotic formulations, and reported outcome measures.

### 2.4 Risk of bias assessment

The risk of bias of the included studies was assessed using the Cochrane Risk of Bias tool (Higgins et al., 2011). Based on the assessment criteria, studies were classified as having low, high, or unclear risk of bias, and methodological quality was graded as follows: Grade A for studies with all domains rated as low risk, Grade B for those with no high-risk domains but at least one unclear domain, and Grade C for studies with any domain rated as high risk. Two reviewers (Z-QH and H-YL) independently conducted all assessments, and disagreements were resolved by consensus or, if needed, by consulting a third reviewer (EZ or B-LS).

### 2.5 Statistical methods

For continuous outcomes, mean differences (MDs) with standard deviations (SDs) were used when measurements shared the same units or methods; otherwise, standardized mean differences (SMDs) were applied. Effect sizes were presented with 95% confidence intervals (CIs). Heterogeneity was assessed using the Cochrane *Q* test and *I*^2^ statistic. A fixed-effects model was used when *I*^2^ < 50% and *p* > 0.10; otherwise, a random-effects model was applied. Subgroup analyses were performed based on probiotic strain combinations and intervention duration, and sensitivity analyses assessed the stability of the results. When at least 10 studies were available for an outcome, funnel plots and Egger’s test were used to evaluate publication bias. All tests were two-sided, with *p* < 0.05 considered statistically significant. Meta-analyses were conducted using R (version 4.5.1).

## 3 Results

### 3.1 Literature selection

**Figure 1:**
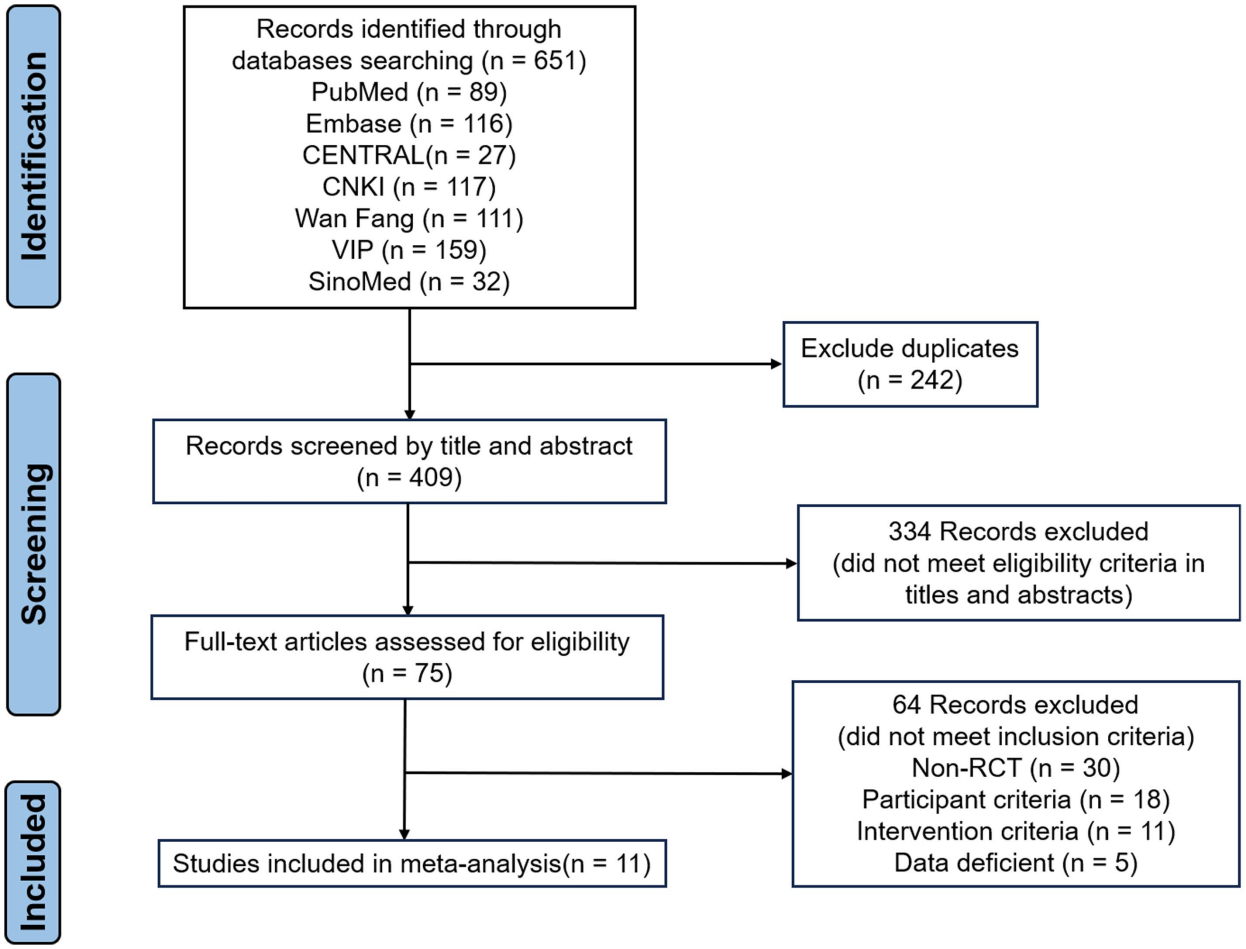
Flow diagram of the literature search and selection process.

A total of 651 records were identified through electronic database searching, and 242 duplicates were removed. Screening of titles and abstracts for the remaining 409 records resulted in the exclusion of 334 irrelevant studies, leaving 75 articles for full-text assessment. After full-text review based on the predefined inclusion and exclusion criteria, 11 studies were finally included. The study selection process is presented in Figure 1.

### 3.2 Basic characteristics of included studies

This review included a total of 11 RCTs (Ni et al., 2013; Abbasi et al., 2017; Soleimani et al., 2017; Mafi et al., 2018; Arani et al., 2019; Miraghajani et al., 2019; Tang et al., 2020; Jiang et al., 2021; Sheng et al., 2023; Zhang et al., 2023; Pingali et al., 2024) involving 726 participants, comprising 390 in the intervention groups and 336 in the control groups. One multi-arm RCT (Ni et al., 2013) included both low-dose and high-dose probiotic intervention arms, which were therefore treated as two independent comparisons. Among the included studies, eight RCTs (Abbasi et al., 2017; Soleimani et al., 2017; Mafi et al., 2018; Arani et al., 2019; Miraghajani et al., 2019; Jiang et al., 2021; Zhang et al., 2023; Pingali et al., 2024) compared probiotics with placebo, and three (Ni et al., 2013; Tang et al., 2020; Sheng et al., 2023) compared probiotics with standard therapy. Three studies (Ni et al., 2013; Abbasi et al., 2017; Pingali et al., 2024) reported mild and transient gastrointestinal discomfort, whereas no serious adverse events were observed in any trial. The key characteristics of the included studies are summarized in Table 1.

**Table 1:** Basic characteristics of included studies.

Study	Source of participants	Sample size (E/C)	Intervention	Comparison	Treatment duration (weeks)	Outcomes
Abbasi et al. (2017)	Iran	20/20	*Lactobacillus plantarum* A7	Regular soy milk	8	a b d
Arani et al. (2019)	Iran	30/30	*Bacillus coagulans* T4	Regular honey	12	a c g i k l m n o p q r
Jiang et al. (2021)	China	42/34	*Bifidobacterium bifidum*, *Lactobacillus acidophilus, Streptococcus thermophilus.*	Placebo capsule	12	b d g h i
Mafi et al. (2018)	Iran	30/30	*Lactobacillus acidophilus*, *Bifidobacterium bifidum*, *Lactobacillus reuteri*, *Lactobacillus fermentum.*	Placebo capsule	12	b c d e g h j k l m n o p q r
Miraghajani et al. (2019)	Iran	20/20	*Lactobacillus plantarum* A7	Regular soy milk	8	f
Ni et al. (2013) (low-dose)	China	18/17	*Paecilomyces hepiali* Cs-4 (2.97 g/d)	Conventional therapy	12	a c e g h k l m n
Ni et al. (2013) (high-dose)	China	17/17	*Paecilomyces hepiali* Cs-4 (5.94 g/d)	Conventional therapy	12	
Pingali et al. (2024)	India	24/23	*Lactobacillus salivarius*, *Lactobacillus casei*, *Lactobacillus plantarum*, *Lactobacillus acidophilus*, *Bifidobacterium breve*, *Bacillus coagulans.*	Placebo capsule	12	g h i k l m n p r
Sheng et al. (2023)	China	58/57	*Bifidobacterium longum*, *Lactobacillus bulgaricus*, *Streptococcus thermophilus.*	Conventional therapy	8	a b c d f g k l m n
Soleimani et al. (2017)	Iran	30/30	*Lactobacillus acidophilus, Lactobacillus casei, Bifidobacterium bifidum.*	Placebo capsule	12	a c d g h i k l m n o p q r
Tang et al. (2020)	China	45/45	*Bifidobacterium longum, Lactobacillus bulgaricus, Streptococcus thermophilus.*	Conventional therapy	12	a c e f g h j m n p q r
Zhang et al. (2023)	China	56/30	*Lactobacillus acidophilus, Lactobacillus casei, Bifidobacterium bifidum.*	Regular soy milk	12	a b d g i p

E, Experimental group; C, Control group; a. Scr; b. eGFR; c. BUN; d. UACR; e. 24 h UP; f. Cys-C; g. FPG; h. HbA1c; i. HOMA-IR; j. 2 h-PG; k. TG; l. TC; m. HDL-C; n. LDL-C; o. hs-CRP; p. MDA; q. TAC; r. NO.

### 3.3 Risk of bias evaluation

**Figure 2:**
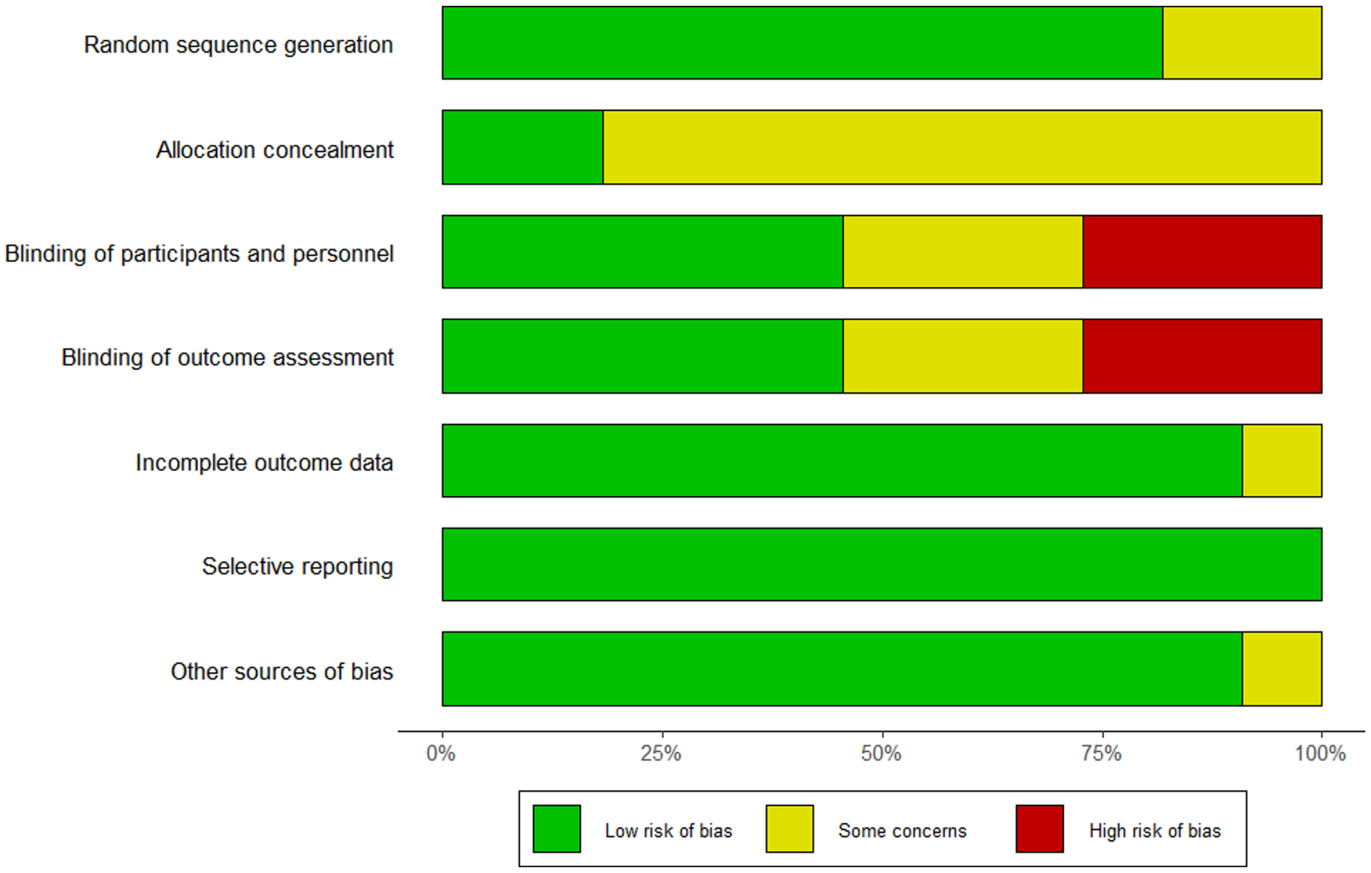
Methodological quality assessment of included studies.

The quality assessment showed that among the included studies, one (Pingali et al., 2024) was rated as Grade A, seven (Abbasi et al., 2017; Soleimani et al., 2017; Mafi et al., 2018; Arani et al., 2019; Miraghajani et al., 2019; Jiang et al., 2021; Zhang et al., 2023) as Grade B, and three (Ni et al., 2013; Tang et al., 2020; Sheng et al., 2023) as Grade C. Nine RCTs (Ni et al., 2013; Soleimani et al., 2017; Mafi et al., 2018; Arani et al., 2019; Miraghajani et al., 2019; Tang et al., 2020; Jiang et al., 2021; Sheng et al., 2023; Pingali et al., 2024) explicitly reported their methods of random sequence generation, of which five (Soleimani et al., 2017; Mafi et al., 2018; Miraghajani et al., 2019; Jiang et al., 2021; Pingali et al., 2024) used computer-generated randomization, three (Ni et al., 2013; Arani et al., 2019; Tang et al., 2020) applied random number tables, and one (Sheng et al., 2023) adopted stratified randomization; an additional two (Abbasi et al., 2017; Zhang et al., 2023) studies mentioned randomization but did not describe the specific method used. Regarding allocation concealment, two (Miraghajani et al., 2019; Pingali et al., 2024) RCTs employed sealed envelopes. In the blinding assessment, five (Abbasi et al., 2017; Soleimani et al., 2017; Mafi et al., 2018; Jiang et al., 2021; Pingali et al., 2024) RCTs were judged to have a low risk of bias due to the use of rigorous double-blind, placebo-controlled designs, whereas three (Ni et al., 2013; Tang et al., 2020; Sheng et al., 2023) were assessed as high risk because blinding was not implemented and no placebo control was used. One RCT (Jiang et al., 2021) had a high attrition rate without reporting the reasons for withdrawal, rendering its impact on outcomes unclear; the remaining studies reported complete outcome data with no evidence of selective reporting. The detailed results are presented in Figure 2.

### 3.4 Meta-analysis results

#### 3.4.1 Renal function markers

**Figure 3:**
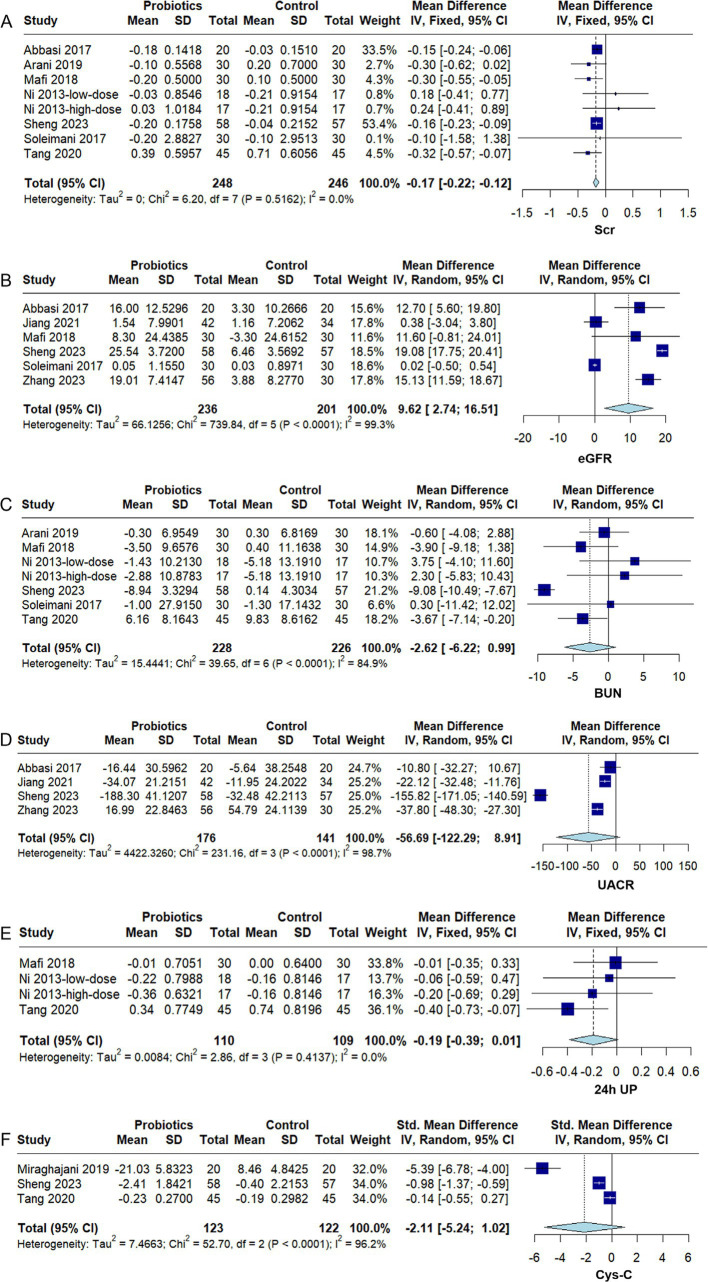
Forest plots of the effect of probiotics on renal function markers in diabetic kidney disease (DKD). **(A)** Serum creatinine (Scr), **(B)** estimated glomerular filtration rate (eGFR), **(C)** blood urea nitrogen (BUN), **(D)** urine albumin-to-creatinine ratio (UACR), **(E)** 24-h urine protein (24 h UP), **(F)** cystatin C (Cys-C). Effect sizes are expressed as mean difference (MD) or standardized mean difference (SMD) with 95% confidence interval (CI). A fixed- or random-effects model was applied according to heterogeneity (*I*^2^). Squares indicate individual studies and diamonds indicate pooled estimates.

Scr levels were reported by seven RCTs (Ni et al., 2013; Abbasi et al., 2017; Soleimani et al., 2017; Mafi et al., 2018; Arani et al., 2019; Tang et al., 2020; Sheng et al., 2023). No significant statistical heterogeneity was detected among the included studies (*I^2^* = 0%, *p* = 0.52). Although the direction of effect varied in some trials, the overall effect was consistent, showing that probiotic supplementation was associated with a statistically significant reduction in Scr (MD − 0.17, 95% CI − 0.22 to −0.12, *p* < 0.0001) (Figure 3A). Subgroup analyses indicated that probiotics effectively reduced Scr levels across different intervention durations and probiotic strain combinations (Supplementary Figure S1).

eGFR levels were reported by six RCTs (Abbasi et al., 2017; Soleimani et al., 2017; Mafi et al., 2018; Jiang et al., 2021; Sheng et al., 2023; Zhang et al., 2023). Substantial statistical heterogeneity was detected among these studies (*I^2^* = 99.3%, *p* < 0.0001). The pooled analysis showed that eGFR was higher in the probiotic group than in the control group following intervention (MD 9.62, 95% CI 2.74 to 16.51, *p* = 0.006) (Figure 3B). Subgroup analysis revealed that the 8-week intervention significantly improved eGFR (MD 16.88, 95% CI 10.94 to 22.82, *p* < 0.05), whereas the 12-week intervention did not yield a statistically significant effect (MD 6.19, 95% CI − 1.82 to 14.19, *p* > 0.05) (Supplementary Figure S1). The statistically significant difference between these subgroups (*p* < 0.05) suggested that intervention duration was a potential source of the observed heterogeneity. Sensitivity analysis indicated that the study by Zhang et al. (2023) had a noticeable influence on the overall effect size of the 12-week intervention. However, excluding this study did not alter the final direction of the overall effect (Supplementary Figure S2).

Six RCTs (Ni et al., 2013; Soleimani et al., 2017; Mafi et al., 2018; Arani et al., 2019; Tang et al., 2020; Sheng et al., 2023) reported BUN levels, and substantial heterogeneity was observed across studies (*I^2^* = 84.9%, *p* < 0.0001). The meta-analysis indicated that probiotic supplementation did not significantly reduce BUN (MD − 2.62, 95% CI − 6.22 to 0.99, *p* = 0.15) (Figure 3C). Subgroup analysis showed that multi-strain formulations significantly decreased BUN levels (MD − 5.49, 95% CI − 9.26 to −1.71, *p* < 0.05), whereas single-strain interventions produced no significant improvement (MD 0.41, 95% CI − 2.56 to 3.37, *p* > 0.05) (Supplementary Figure S1). Sensitivity analysis indicated that the study by Sheng et al. (2023) had a notable influence on the overall effect estimate for multi-strain probiotics, and exclusion of this study markedly reduced overall heterogeneity (Supplementary Figure S2).

Four RCTs (Abbasi et al., 2017; Jiang et al., 2021; Sheng et al., 2023; Zhang et al., 2023) reported UACR levels, and substantial heterogeneity was observed among the studies (*I^2^* = 98.7%, *p* < 0.0001). The meta-analysis indicated that probiotic supplementation did not lead to a significant improvement in UACR (MD − 56.69, 95% CI − 122.29 to 8.91, *p* = 0.09) (Figure 3D). Subgroup analysis suggested that a 12-week probiotic intervention significantly reduced UACR (MD − 29.94, 95% CI − 45.30 to −14.57, *p* < 0.05), whereas an 8-week intervention did not result in notable improvement (MD − 83.52, 95% CI − 225.63 to 58.60, *p* > 0.05) (Supplementary Figure S1).

Three RCTs (Ni et al., 2013; Mafi et al., 2018; Tang et al., 2020) reported 24 h UP levels, and no significant heterogeneity was detected among the studies (*I^2^* = 0%, *p* = 0.41). The meta-analysis indicated that probiotic supplementation did not markedly reduce 24 h UP (MD − 0.19, 95% CI − 0.39 to 0.01, *p* = 0.41) (Figure 3E). Subgroup analyses further showed that probiotic strain combinations did not exert a significant moderating effect on the impact of probiotics on 24 h UP (Supplementary Figure S1).

Three RCTs (Miraghajani et al., 2019; Tang et al., 2020; Sheng et al., 2023) reported Cys-C levels, and substantial heterogeneity was observed among the studies (*I^2^* = 96.2%, *p* < 0.0001). The meta-analysis indicated that probiotic supplementation did not lead to a significant improvement in Cys-C (SMD −2.11, 95% CI −5.24 to 1.02, *p* = 0.19) (Figure 3F). Sensitivity analysis showed that no individual study substantially affected the stability of the overall effect estimate (Supplementary Figure S2).

#### 3.4.2 Glycemic metabolism markers

**Figure 4:**
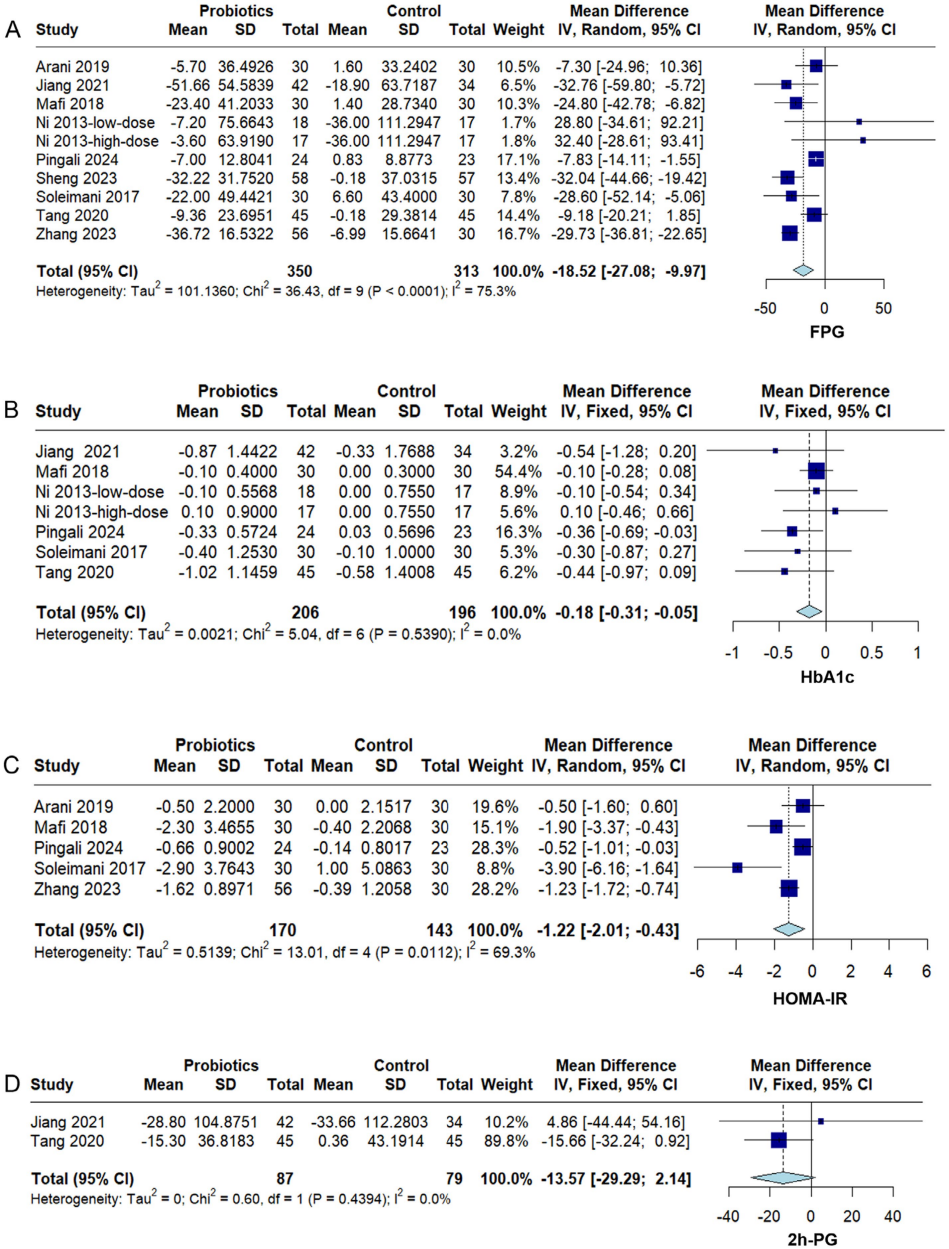
Forest plots of the effect of probiotics on glucose metabolism markers in diabetic kidney disease (DKD). **(A)** Fasting plasma glucose (FPG), **(B)** glycated hemoglobin (HbA1c), **(C)** homeostatic model assessment for insulin resistance (HOMA-IR), **(D)** 2-h postprandial glucose (2 h-PG). Effect sizes are expressed as mean difference (MD) or standardized mean difference (SMD) with 95% confidence interval (CI). A fixed- or random-effects model was applied according to heterogeneity (*I*^2^). Squares indicate individual studies and diamonds indicate pooled estimates.

Nine RCTs (Ni et al., 2013; Soleimani et al., 2017; Mafi et al., 2018; Arani et al., 2019; Tang et al., 2020; Jiang et al., 2021; Sheng et al., 2023; Zhang et al., 2023; Pingali et al., 2024) reported FPG levels, and substantial heterogeneity was identified across studies (*I^2^* = 75.3%, *p* < 0.0001). The pooled analysis demonstrated that probiotic supplementation significantly reduced FPG (MD − 18.52, 95% CI − 27.08 to −9.97, *p* < 0.0001) (Figure 4A). Subgroup analysis indicated that multi-strain probiotics significantly lowered FPG (MD − 21.83, 95% CI − 30.96 to −12.69, *p* < 0.05), whereas single-strain probiotics showed no significant improvement (MD 5.79, 95% CI − 23.05 to 34.63, *p* > 0.05) (Supplementary Figure S1). Sensitivity analyses confirmed the robustness of the overall effect estimate, with no single study exerting a disproportionate influence (Supplementary Figure S2).

Six RCTs (Ni et al., 2013; Soleimani et al., 2017; Mafi et al., 2018; Tang et al., 2020; Jiang et al., 2021; Pingali et al., 2024) reported HbA1c levels, and no significant heterogeneity was detected among the studies (*I^2^* = 0%, *p* = 0.54). Probiotic supplementation significantly reduced HbA1c (MD −0.18, 95% CI −0.31 to −0.05, *p* = 0.009) (Figure 4B). Subgroup analysis showed that multi-strain probiotic formulations significantly improved HbA1c (MD −0.20, 95% CI −0.35 to −0.06, *p* < 0.05), whereas no significant effect was observed for single-strain interventions (MD − 0.02, 95% CI − 0.37 to 0.32, *p* > 0.05) (Supplementary Figure S1).

Five RCTs (Soleimani et al., 2017; Mafi et al., 2018; Arani et al., 2019; Zhang et al., 2023; Pingali et al., 2024) reported HOMA-IR levels, and substantial heterogeneity was present across studies (*I^2^* = 69.3%, *p* = 0.01). Probiotic supplementation significantly reduced HOMA-IR (MD − 1.22, 95% CI − 2.01 to −0.43, *p* = 0.002) (Figure 4C). Sensitivity analyses indicated that the pooled result was robust, as exclusion of any single study did not alter the direction of the overall effect (Supplementary Figure S2).

Two RCTs (Tang et al., 2020; Jiang et al., 2021) reported 2 h-PG levels, and no significant heterogeneity was observed between the studies (*I^2^* = 0%, *p* = 0.44). The pooled results indicated that probiotic supplementation did not significantly reduce 2 h-PG levels (MD − 13.57, 95% CI − 29.29 to 2.14, *p* = 0.09) (Figure 4D).

#### 3.4.3 Lipid metabolism markers

**Figure 5:**
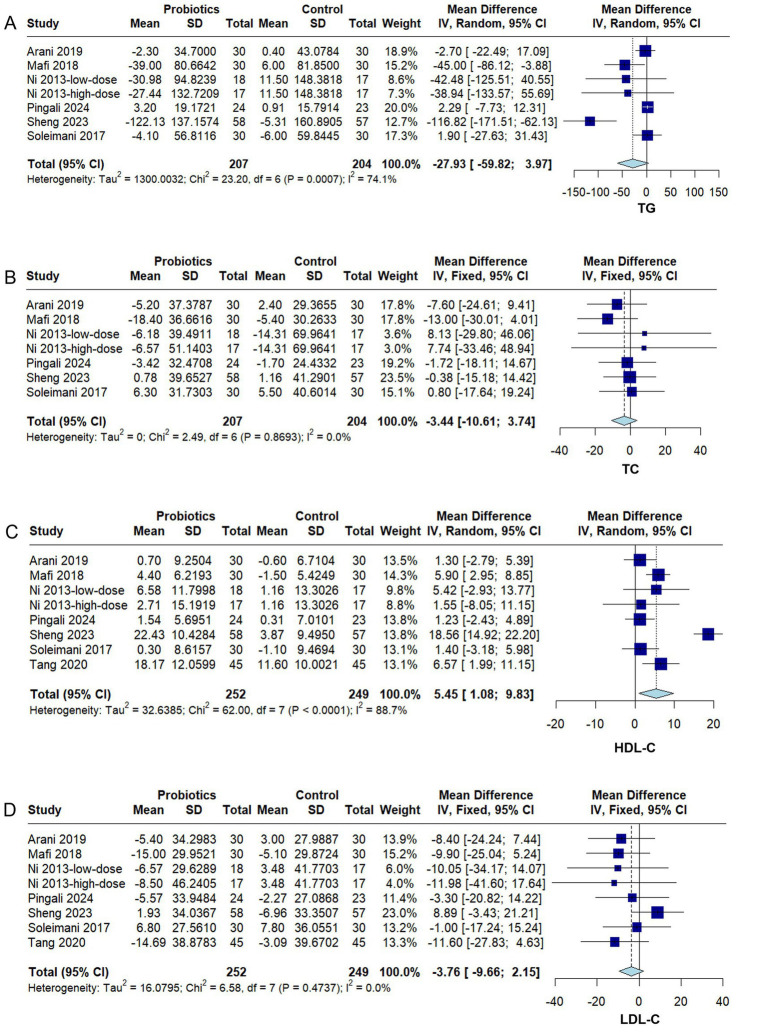
Forest plots of the effect of probiotics on lipid metabolism markers in diabetic kidney disease (DKD). **(A)** Triglycerides (TG), **(B)** total cholesterol (TC), **(C)** high-density lipoprotein cholesterol (HDL-C), **(D)** low-density lipoprotein cholesterol (LDL-C). Effect sizes are expressed as mean difference (MD) or standardized mean difference (SMD) with 95% confidence interval (CI). A fixed- or random-effects model was applied according to heterogeneity (*I*^2^). Squares indicate individual studies and diamonds indicate pooled estimates.

Six RCTs (Ni et al., 2013; Soleimani et al., 2017; Mafi et al., 2018; Miraghajani et al., 2019; Sheng et al., 2023; Pingali et al., 2024) reported TG and TC levels. Significant heterogeneity was observed among studies for TG (*I^2^* = 74.1%, *p* = 0.0007), whereas no significant heterogeneity was detected for TC (*I^2^* = 0%, *p* = 0.87). The meta-analysis indicated that probiotic supplementation did not significantly reduce TG (MD − 27.93, 95% CI − 59.82 to 3.97, *p* = 0.07) (Figure 5A) or TC (MD − 3.44, 95% CI − 10.61 to 3.74, *p* = 0.35) (Figure 5B). Subgroup analyses based on probiotic strain combinations revealed no statistically significant differences for either TG or TC (Supplementary Figure S1). Sensitivity analysis further demonstrated that, for TG, exclusion of any single study did not alter the direction of the effect observed for multi-strain probiotic supplementation (Supplementary Figure S2).

Seven RCTs (Ni et al., 2013; Soleimani et al., 2017; Mafi et al., 2018; Arani et al., 2019; Tang et al., 2020; Sheng et al., 2023; Pingali et al., 2024) reported HDL-C and LDL-C levels. Significant heterogeneity was observed among studies for HDL-C (*I^2^* = 88.7%, *p* < 0.0001), whereas no significant heterogeneity was detected for LDL-C (*I^2^* = 0%, *p* = 0.47). The meta-analysis showed that probiotic supplementation significantly increased HDL-C (MD 5.45, 95% CI 1.08 to 9.83, *p* = 0.01) (Figure 5C), but did not produce a significant improvement in LDL-C (MD − 3.76, 95% CI − 9.66 to 2.15, *p* = 0.21) (Figure 5D). Subgroup analysis indicated that multi-strain probiotic supplementation significantly improved HDL-C (MD 6.77, 95% CI 0.55 to 12.99, *p* < 0.05) (Supplementary Figure S1), and subsequent sensitivity analysis confirmed the robustness of this subgroup effect (Supplementary Figure S2). However, for LDL-C, no statistically significant effects were observed across probiotic strain subgroups (Supplementary Figure S1).

#### 3.4.4 Inflammation and oxidative stress markers

**Figure 6:**
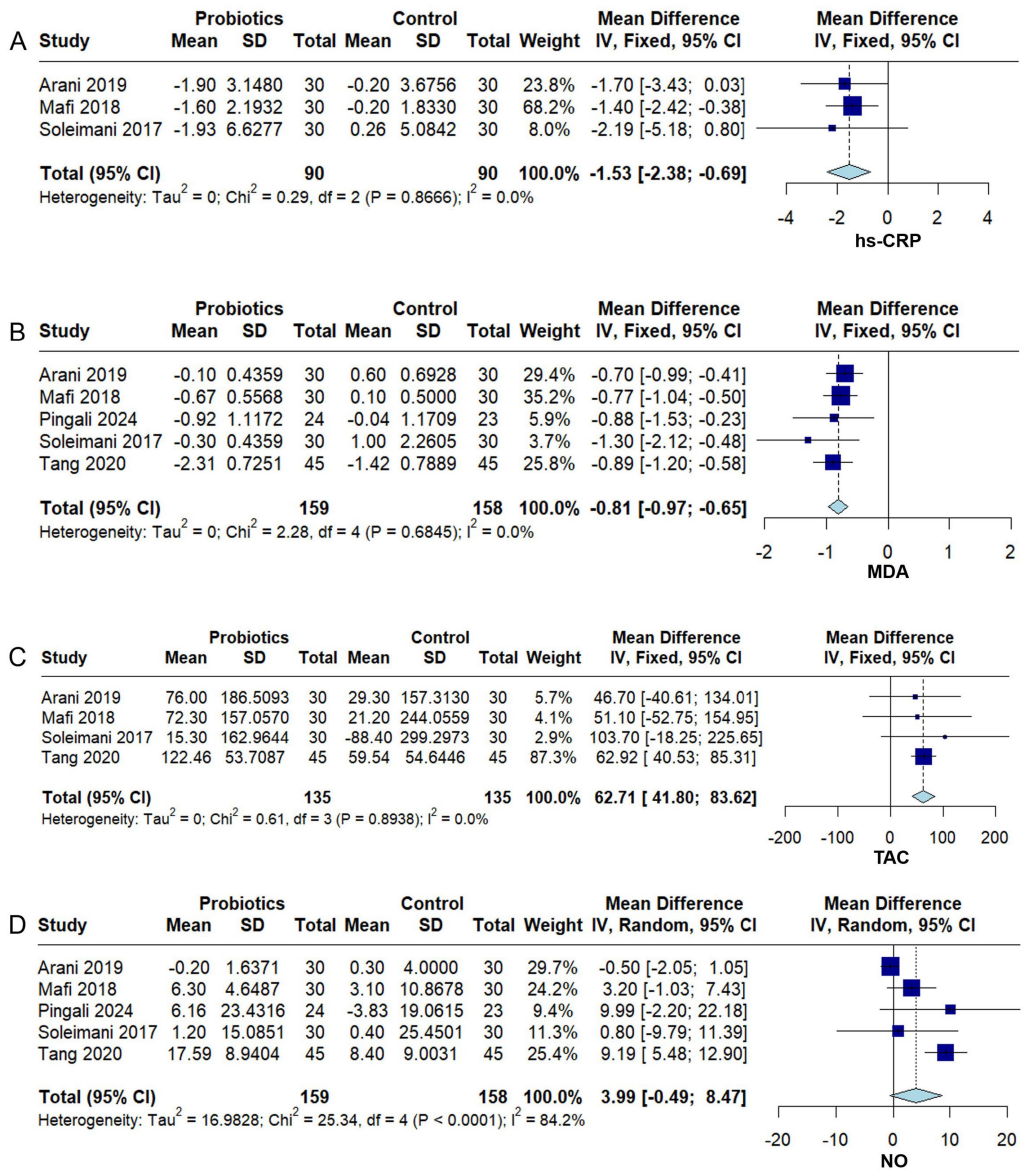
Forest plots of the effect of probiotics on inflammation and oxidative stress markers in diabetic kidney disease (DKD). **(A)** High-sensitivity C-reactive protein (hs-CRP), **(B)** Malondialdehyde (MDA), **(C)** Total antioxidant capacity (TAC), **(D)** Nitric oxide (NO). Effect sizes are expressed as mean difference (MD) or standardized mean difference (SMD) with 95% confidence interval (CI). A fixed- or random-effects model was applied according to heterogeneity (*I*^2^). Squares indicate individual studies and diamonds indicate pooled estimates.

In the pooled analyses of hs-CRP (Soleimani et al., 2017; Mafi et al., 2018; Arani et al., 2019), MDA (Soleimani et al., 2017; Mafi et al., 2018; Arani et al., 2019; Tang et al., 2020; Zhang et al., 2023; Pingali et al., 2024), and TAC (Soleimani et al., 2017; Mafi et al., 2018; Arani et al., 2019; Tang et al., 2020), no significant heterogeneity was observed across studies (*I^2^* = 0%, *p* > 0.10). The overall results showed that probiotic supplementation significantly reduced hs-CRP (MD −1.53, 95% CI −2.38 to −0.69, *p* = 0.0004) (Figure 6A) and MDA (MD −0.81, 95% CI −0.97 to −0.65, *p* < 0.0001) (Figure 6B), while significantly increasing TAC (MD 62.71, 95% CI 41.80 to 83.62, *p* < 0.0001) (Figure 6C).

In the pooled analysis of NO (Soleimani et al., 2017; Mafi et al., 2018; Arani et al., 2019; Tang et al., 2020; Pingali et al., 2024), substantial heterogeneity was detected among the studies (*I^2^* = 84.2%, *p* < 0.0001). The meta-analysis indicated that probiotic supplementation did not significantly improve NO levels (MD 3.99, 95% CI − 0.49 to 8.47, *p* = 0.08) (Figure 6D). However, sensitivity analysis revealed limited robustness of the overall effect estimate, which was primarily influenced by two studies—Arani et al. (2019) and Tang et al. (2020) (Supplementary Figure S2). Specifically, exclusion of Arani et al. (2019) resulted in a statistically significant pooled effect (MD 5.99, 95% CI 1.65 to 10.32, *p* = 0.007), whereas exclusion of Tang et al. (2020) yielded a nonsignificant effect, suggesting minimal improvement in NO with probiotic supplementation (MD 1.44, 95% CI − 1.88 to 4.76, *p* = 0.39).

### 3.5 Publication bias

In this Meta-analysis, only FPG met the criteria for publication bias assessment. The Egger’s linear regression test indicated no significant publication bias for the FPG outcome (Supplementary Figure S3).

## 4 Discussion

Dysregulation of the gut microbiota is closely linked to the onset and progression of DKD, and probiotics, as an important microbiome-modulating approach, may exert regulatory effects through their influence on the gut-kidney axis. Available evidence suggests that probiotics may exert renoprotective effects in DKD by remodeling the gut microbiota, enriching beneficial taxa such as *Bifidobacterium* and *Lactobacillus*, promoting SCFAs production, and reducing the generation and translocation of gut-derived toxins, including IS, *p*CS, and TMAO (Huang and Chen, 2024). These changes may help restore intestinal barrier integrity, mitigate systemic inflammation and metabolic dysfunction, and ultimately slow DKD progression. Guided by these mechanistic insights, this study synthesised evidence from available RCTs and quantitatively assessed the overall effects of probiotic supplementation in patients with DKD. Our meta-analysis indicates that probiotics may provide benefits in improving glycemic and lipid disturbances, enhancing renal outcomes and reducing systemic inflammation and oxidative stress.

We summarized 11 RCTs investigating the benefits of probiotics in patients with DKD, including four trials using single-strain formulations and seven using multi-strain combinations. The study populations encompassed both non-dialysis patients with CKD stages 3–5 and patients undergoing dialysis. Across these interventions, a total of 13 probiotic strains were utilized. *Lactobacillus* and *Bifidobacterium* species were the most frequently used, with *Lactobacillus strains* included in nine studies, namely *Lactobacillus acidophilus*, *Lactobacillus sali*var*ius*, *Lactobacillus casei*, *Lactobacillus bulgaricus*, *Lactobacillus reuteri*, *Lactobacillus fermentum*, and *Lactobacillus plantarum*, and *Bifidobacterium* strains used in seven studies, including *Bifidobacterium breve*, *Bifidobacterium longum*, and *Bifidobacterium bifidum*. Additional strains included *Streptococcus thermophilus*, *Paecilomyces hepiali*, and *Bacillus coagulans*. The duration of probiotic supplementation varied across studies, with three trials administering probiotics for 8 weeks and eight trials for 12 weeks. Given that differences in strain composition and intervention duration may influence the effect estimates, our meta-analysis accounted for these potential sources of heterogeneity and evaluated the robustness of pooled outcomes through sensitivity analyses.

We first evaluated the effects of probiotic supplementation on renal function in patients with DKD. Scr is a traditional marker reflecting glomerular filtration function, and our findings showed that probiotic supplementation significantly reduced Scr levels in DKD patients under both single-strain and multi-strain interventions. Notably, the duration of supplementation appeared to influence improvements in eGFR. An eight-week intervention produced a significant benefit, whereas a 12-week intervention did not yield a comparable effect. This observation may suggest that the improvement in eGFR attributed to probiotics primarily reflects short-term functional changes. It may also be influenced by patient heterogeneity, concomitant medication use, or differences in study design. In addition, multi-strain formulations showed a significant advantage in reducing BUN, whereas no comparable improvement was observed with single-strain interventions. This pattern is biologically plausible, as combinations of strains typically exhibit enhanced colonization capacity and synergistic metabolic activity (Kwoji et al., 2021). We also observed that probiotic supplementation for 12 weeks resulted in a significant reduction in UACR, yet no appreciable effects were detected for 24 h UP or Cys-C. These findings indicate that the observed benefits of probiotic supplementation may be largely confined to short-term functional improvements, while evidence for effects on structural renal injury remains insufficient. Therefore, its renoprotective effects should be interpreted with caution.

Subsequently, we further examined the effects of probiotic supplementation on glucose and lipid metabolism in patients with DKD. Quantitative analyses of four glycemic indicators showed that probiotics significantly reduced FPG, HbA1c and HOMA-IR, suggesting beneficial effects on long-term glycemic control and insulin sensitivity. These findings are highly consistent with previous meta-analyses conducted in individuals with type 2 diabetes (T2DM) (Paul et al., 2022). Preclinical studies also support this view. Probiotics containing *Lactobacillus* strains have been shown to improve glucose metabolism in T2DM mice through multiple metabolic pathways, including modulation of glucagon-like peptide-1 (GLP-1) secretion, reductions in fasting glucose, enhancement of insulin release and upregulation of glucose transporters (Song et al., 2023). Other studies have reported that *Bifidobacterium* increases intestinal levels of indole derivatives in diabetic mouse models, thereby activating the AHR/GLP-1/ pancreatic and duodenal homeobox 1 (PDX-1) signaling pathway, promoting GLP-1 secretion and improving islet function (Chen J. et al., 2025). These mechanistic observations provide biological support for the glycometabolic improvements associated with probiotic supplementation. It is important to note that we did not observe a significant effect of probiotics on 2 h-PG levels in patients with DKD, although this result should be interpreted with caution. Only two RCTs contributed data for this outcome, resulting in limited statistical power, and 2 h-PG is influenced by multiple physiological factors, including gastric emptying and intestinal glucose absorption, leading to considerably greater variability than FPG or HbA1c. Despite these limitations, the overall evidence indicates that probiotic supplementation confers clear metabolic benefits for glucose regulation in patients with DKD.

Dyslipidaemia is one of the most common metabolic complications in patients with DKD and is associated with a substantially increased risk of cardiovascular events (Zhu et al., 2025). In our analysis, probiotic supplementation was associated with a significant increase in HDL-C concentrations, whereas no clear improvements were observed in TG, TC or LDL-C. These findings differ partly from those reported in previous meta-analyses in T2DM and may reflect the distinctive lipid metabolism disturbances seen in DKD (Bock et al., 2021). Previous evidence suggests that chronic inflammation and the accumulation of uraemic toxins in CKD can suppress the activities of lipoprotein lipase and hepatic lipase, resulting in impaired clearance of very-low-density lipoprotein and its remnants (Vaziri, 2006). These disturbances are unlikely to be corrected through short-term probiotic intervention. Furthermore, the widespread use of lipid-lowering therapy among patients with DKD lowers baseline lipid concentrations and may reduce the likelihood of detecting additional lipid-modifying effects attributable to probiotics. Future studies in more homogeneous populations with standardised lipid-lowering regimens are needed to determine the potential role of probiotic supplementation in lipid management in CKD.

Chronic inflammation and oxidative stress are central drivers of DKD progression and are closely linked to disturbances in the gut microbiota (Mosterd et al., 2021). Gut dysbiosis can promote intestinal inflammation and impair barrier integrity, facilitating the entry of lipopolysaccharide and gut-derived toxic metabolites, including IS, *p*CS, and TMAO, into the circulation. These changes activate proinflammatory pathways, increase release of inflammatory mediators, and exacerbate oxidative kidney injury. At the same time, depletion of beneficial metabolites, particularly SCFAs, further weakens intestinal barrier homeostasis, immune regulation, and antioxidant defenses (Li et al., 2024). Together, these disturbances create a self-perpetuating cycle of microbial imbalance, metabolic dysfunction, inflammation, and oxidative stress that drives progression of DKD. In our synthesis of data with low heterogeneity, probiotic supplementation was associated with significant reductions in serum hs-CRP and MDA, alongside an increase in TAC, findings that are consistent with those of a previous meta-analysis (Chen X. et al., 2025). Similar anti-inflammatory and antioxidant effects have been reported in populations with childhood obesity (Ding et al., 2025) and in patients with knee osteoarthritis (Tian et al., 2025), suggesting that these benefits may extend across different clinical contexts. However, no significant effect on NO levels was observed in patients with DKD, and this outcome was accompanied by substantial heterogeneity. A plausible explanation relates to the dual biological role of NO. Under physiological conditions, NO supports vasodilation and endothelial homeostasis, whereas in pathological states it rapidly reacts with superoxide to generate peroxynitrite, a potent oxidant that contributes to endothelial dysfunction and renal injury (Wang and He, 2024). This shift from a protective mediator to a damaging species may render NO highly sensitive to differences in oxidative stress burden and probiotic regimens across studies, leading to inconsistent findings. Overall, the available evidence suggests that probiotic supplementation confers meaningful benefits in attenuating chronic inflammation and oxidative stress in patients with DKD.

## 5 Strengths and limitations

This study included only RCTs, providing a relatively high level of evidence for evaluating the true effects of probiotic supplementation in patients with DKD. We further explored potential sources of heterogeneity, including strain composition and intervention duration, through subgroup and sensitivity analyses, which supported the robustness of the main findings. By synthesizing data from 11 RCTs, this review offers an integrated assessment across multiple domains, including renal function, glycemic and lipid metabolism, and inflammatory and oxidative stress markers, thereby providing a more comprehensive evidence base for understanding the effects of probiotics in DKD. Nevertheless, several limitations should be acknowledged. The composition of the gut microbiota varies substantially between individuals and is influenced by genetics, diet and environmental factors, which may affect the generalisability of the findings. In addition, the included trials differed considerably in their intervention formulations, and detailed analyses of specific strains or dose–response relationships were not possible, limiting further investigation into strain-specific mechanisms. Moreover, some key outcomes, such as Cys-C and 2 h-PG, were reported in only a small number of studies and showed considerable heterogeneity, requiring cautious interpretation of these effect estimates. These factors may constrain the precision with which the effects of probiotic therapy can be evaluated.

## 6 Conclusion

The findings of this study suggest that probiotic supplementation may offer benefits in improving metabolic disturbances, renal function and systemic inflammation and oxidative stress in patients with DKD. These results provide a basis for further investigation into the potential role of probiotics in the management of DKD.

## Data Availability

The original contributions presented in the study are included in the article/Supplementary material, further inquiries can be directed to the corresponding authors.
