# Supplementary material for: Effects of probiotic supplementation on diabetic kidney disease: a systematic review and meta-analysis of randomized controlled trials
Source: Front Microbiol. 2026 May 20;17:1760954. doi: 10.3389/fmicb.2026.1760954 (PMC13230064; doi:10.3389/fmicb.2026.1760954)
Supplement: Supplementary file 1 [file Table_1.DOCX]

Supplementary Table S1 Search strategy

| **Table 1. Search strategy used in PubMed database** | |
| --- | --- |
| order | search terms |
| #1 | "Diabetic Nephropathies"[Mesh] |
| #2 | ((((((((((((((((Nephropathies, Diabetic) OR (Nephropathy, Diabetic)) OR (Diabetic Kidney Disease)) OR (Diabetic Kidney Diseases)) OR (Kidney Disease, Diabetic)) OR (Kidney Diseases, Diabetic)) OR (Diabetic Nephropathy)) OR (Diabetic Glomerulosclerosis)) OR (Glomerulosclerosis, Diabetic)) OR (Intracapillary Glomerulosclerosis)) OR (Kimmelstiel-Wilson Disease)) OR (Kimmelstiel Wilson Disease)) OR (Nodular Glomerulosclerosis)) OR (Glomerulosclerosis, Nodular)) OR (Kimmelstiel-Wilson Syndrome)) OR (Kimmelstiel Wilson Syndrome)) OR (Syndrome, Kimmelstiel-Wilson) |
| #3 | #1 OR #2 |
| #4 | "Probiotics"[Mesh] |
| #5 | Probiotic |
| #6 | #4 OR #5 |
| #7 | #3 AND #6 |

| **Table 2. Search strategy used in Embase database** | |
| --- | --- |
| order | search terms |
| #1 | 'diabetic nephropathy'/exp |
| #2 | nephropathies, diabetic':ab,ti OR 'nephropathy, diabetic':ab,ti OR 'diabetic kidney disease':ab,ti OR 'diabetic kidney diseases':ab,ti OR 'kidney disease, diabetic':ab,ti OR 'kidney diseases, diabetic':ab,ti OR 'diabetic nephropathies':ab,ti OR 'diabetic glomerulosclerosis':ab,ti OR 'glomerulosclerosis, diabetic':ab,ti OR 'intracapillary glomerulosclerosis':ab,ti OR 'kimmelstiel-wilson disease':ab,ti OR 'kimmelstiel wilson disease':ab,ti OR 'nodular glomerulosclerosis':ab,ti OR 'glomerulosclerosis, nodular':ab,ti OR 'kimmelstiel-wilson syndrome':ab,ti OR 'kimmelstiel wilson syndrome':ab,ti OR 'syndrome, kimmelstiel-wilson':ab,ti |
| #3 | #1 OR #2 |
| #4 | 'probiotic agent'/exp |
| #5 | #3 AND #4 |

| **Table 3. Search strategy used in Cochrane library database** | |
| --- | --- |
| order | search terms |
| #1 | MeSH descriptor: [Diabetic Nephropathies] explode all trees |
| #2 | (Nephropathies, Diabetic):ti,ab,kw OR (Nephropathy, Diabetic):ti,ab,kw OR (Diabetic Kidney Disease):ti,ab,kw OR (Diabetic Kidney Diseases):ti,ab,kw OR (Kidney Disease, Diabetic):ti,ab,kw OR (Kidney Diseases, Diabetic):ti,ab,kw OR (Diabetic Nephropathy):ti,ab,kw OR (Diabetic Glomerulosclerosis):ti,ab,kw OR (Glomerulosclerosis, Diabetic):ti,ab,kw OR (Intracapillary Glomerulosclerosis):ti,ab,kw OR (Kimmelstiel-Wilson Disease):ti,ab,kw OR (Kimmelstiel Wilson Disease):ti,ab,kw OR (Nodular Glomerulosclerosis):ti,ab,kw OR (Glomerulosclerosis, Nodular):ti,ab,kw OR (Kimmelstiel-Wilson Syndrome):ti,ab,kw OR (Kimmelstiel Wilson Syndrome):ti,ab,kw OR (Syndrome, Kimmelstiel-Wilson):ti,ab,kw |
| #3 | #1 OR #2 |
| #4 | MeSH descriptor: [Probiotics] explode all trees |
| #5 | (Probiotic):ti,ab,kw |
| #6 | #4 OR #5 |
| #7 | #3 AND #6 |
