# Supplementary material for: Effects of probiotic supplementation on diabetic kidney disease: a systematic review and meta-analysis of randomized controlled trials
Source: Front Microbiol. 2026 May 20;17:1760954. doi: 10.3389/fmicb.2026.1760954 (PMC13230064; doi:10.3389/fmicb.2026.1760954)

Supplementary Figure 1A. Forest plot depicting mean differences (MD) and the 95% confidence interval (CI) for the impact of probiotic supplementation on serum creatinine (Scr) levels according to intervention duration (8 weeks or 12 weeks).


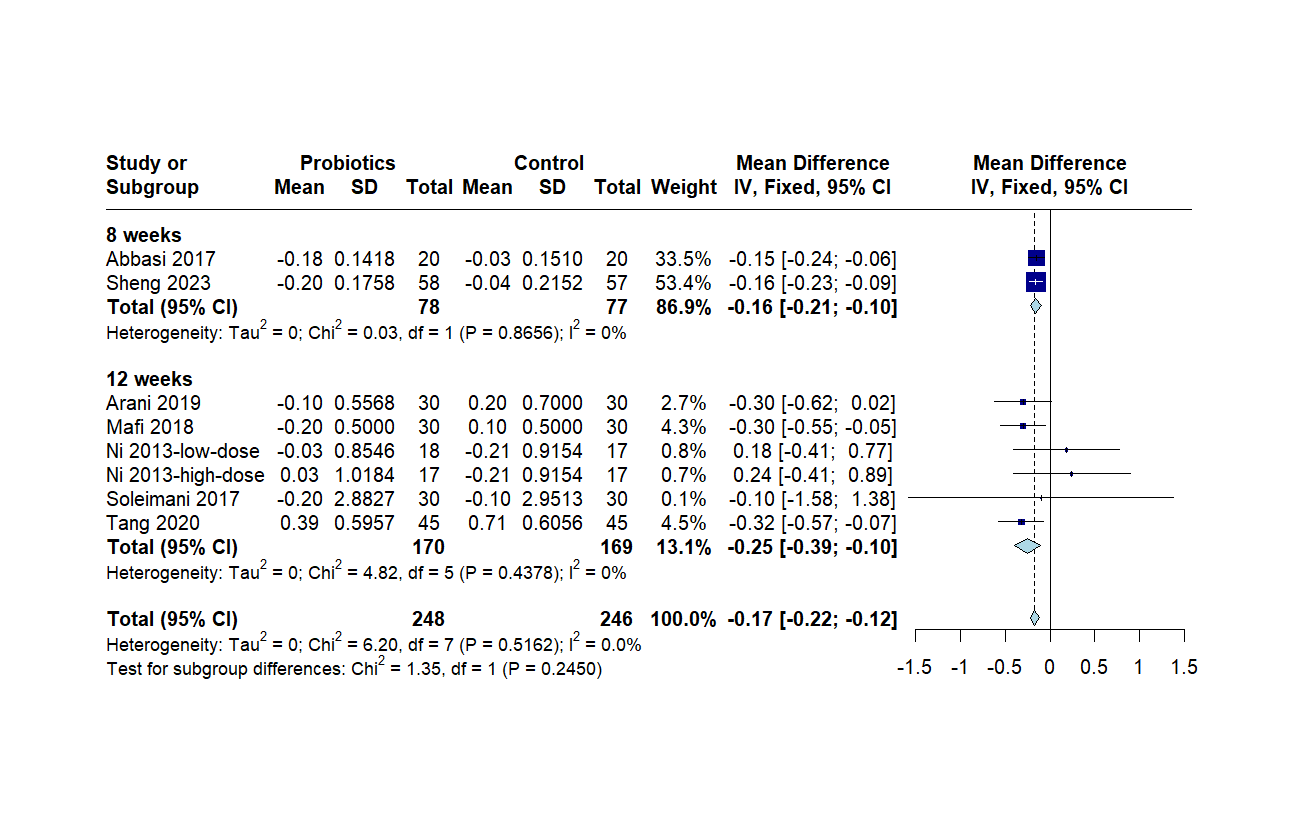


Supplementary Figure 1B. Forest plot depicting mean differences (MD) and the 95% confidence interval (CI) for the impact of probiotic supplementation on estimated glomerular filtration rate (eGFR) levels according to intervention duration (8 weeks or 12 weeks).


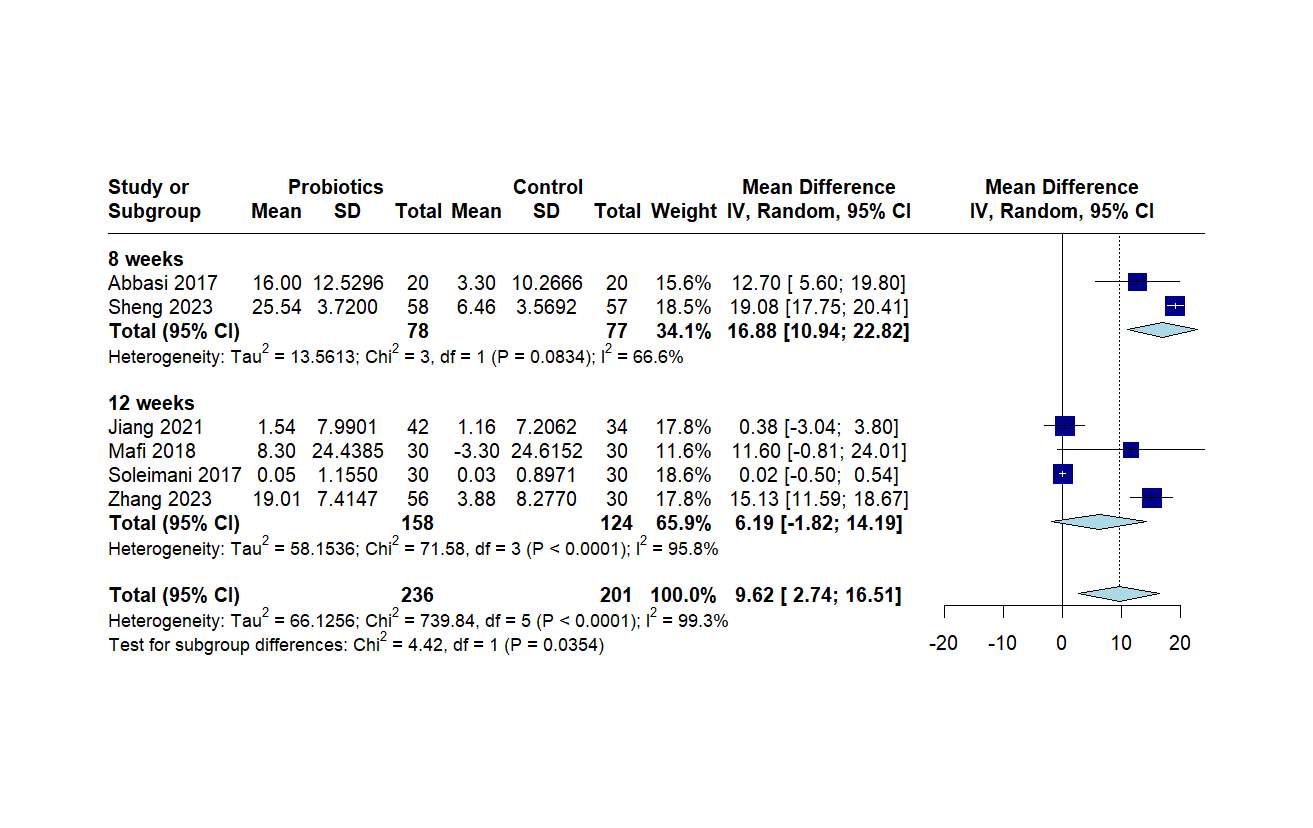


Supplementary Figure 1C. Forest plot depicting mean differences (MD) and the 95% confidence interval (CI) for the impact of probiotic supplementation on urinary albumin-to-creatinine ratio (UACR) levels according to intervention duration (8 weeks or 12 weeks).


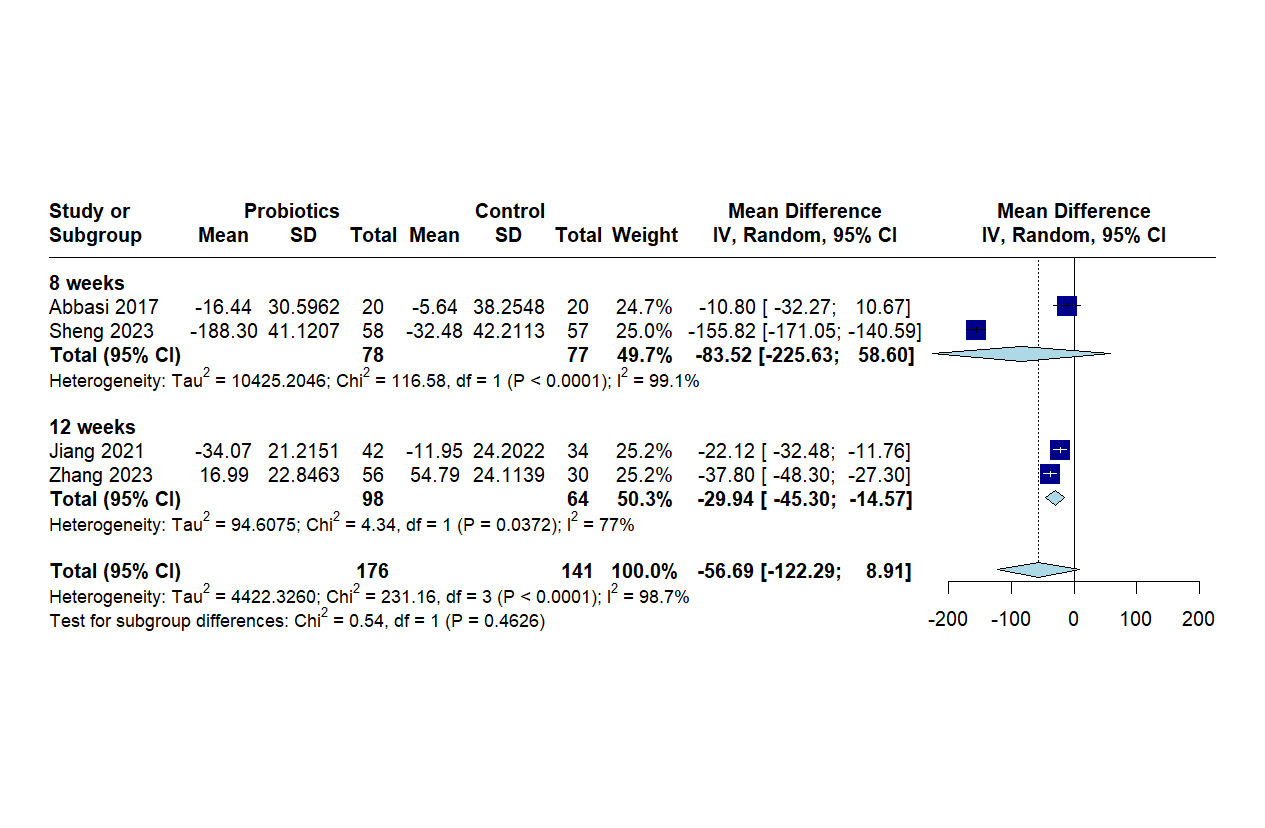


Supplementary Figure 2A. Forest plot depicting mean differences (MD) and the 95% confidence interval (CI) for the impact of probiotic supplementation on serum creatinine (Scr) levels according to strain composition (single-strain or multi-strain).


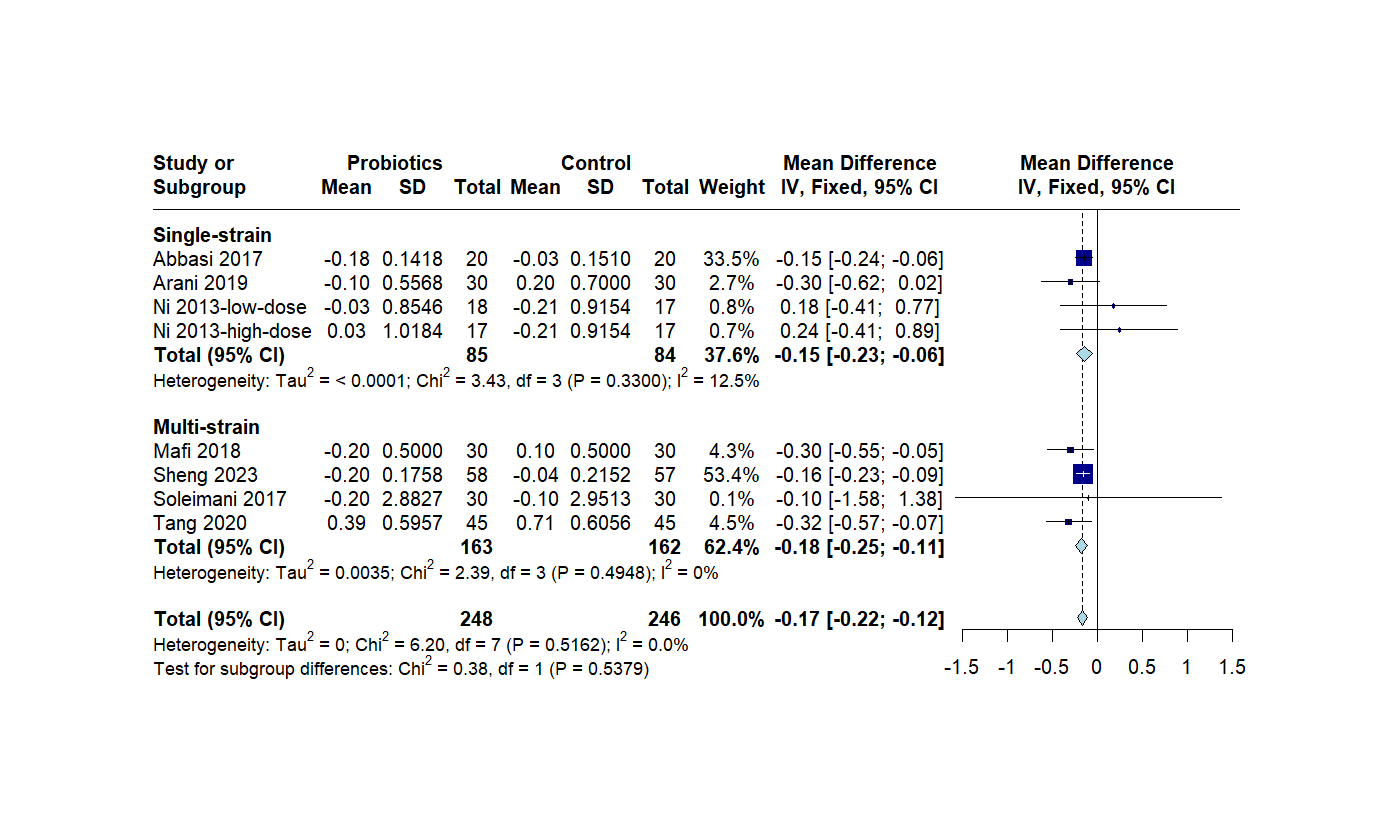


Supplementary Figure 2B. Forest plot depicting mean differences (MD) and the 95% confidence interval (CI) for the impact of probiotic supplementation on blood urea nitrogen (BUN) levels according to strain composition (single-strain or multi-strain).


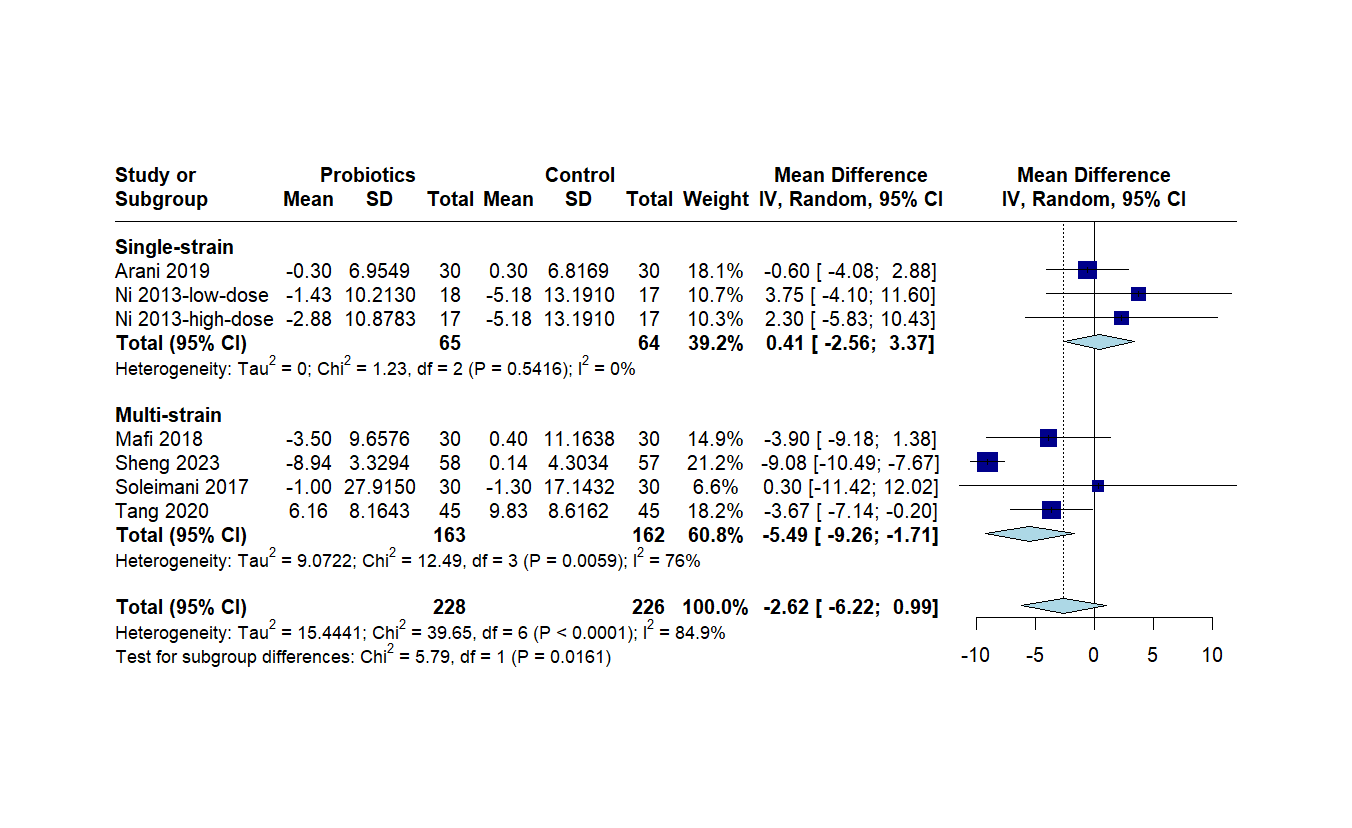


Supplementary Figure 2C. Forest plot depicting mean differences (MD) and the 95% confidence interval (CI) for the impact of probiotic supplementation on 24-hour urine protein quantification (24h UP) levels according to strain composition (single-strain or multi-strain).


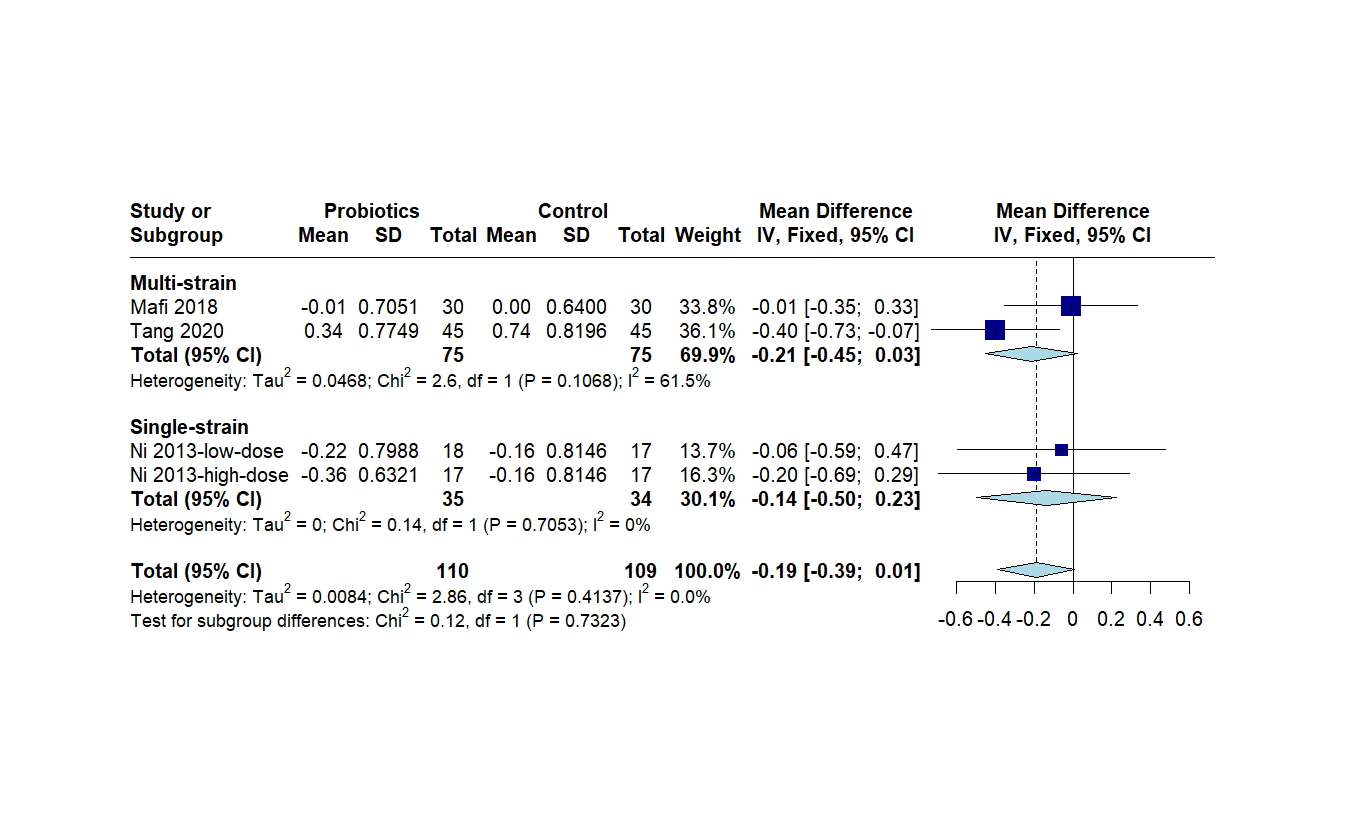


Supplementary Figure 2D. Forest plot depicting mean differences (MD) and the 95% confidence interval (CI) for the impact of probiotic supplementation on glucose metabolism indicators: fasting plasma glucose (FPG) levels according to strain composition (single-strain or multi-strain).


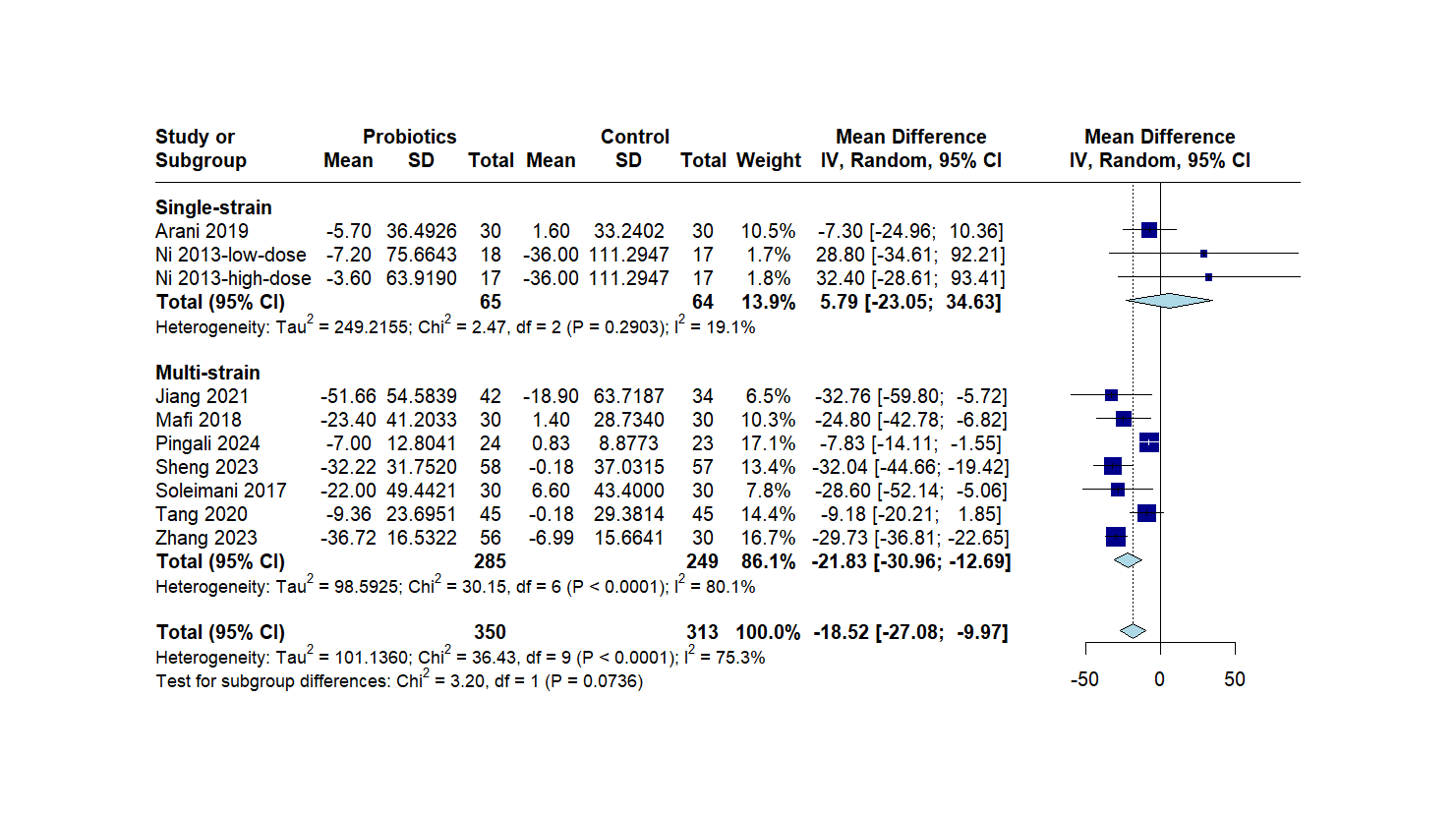


Supplementary Figure 2E. Forest plot depicting mean differences (MD) and the 95% confidence interval (CI) for the impact of probiotic supplementation on glycated hemoglobin (HbA1c) levels according to strain composition (single-strain or multi-strain).


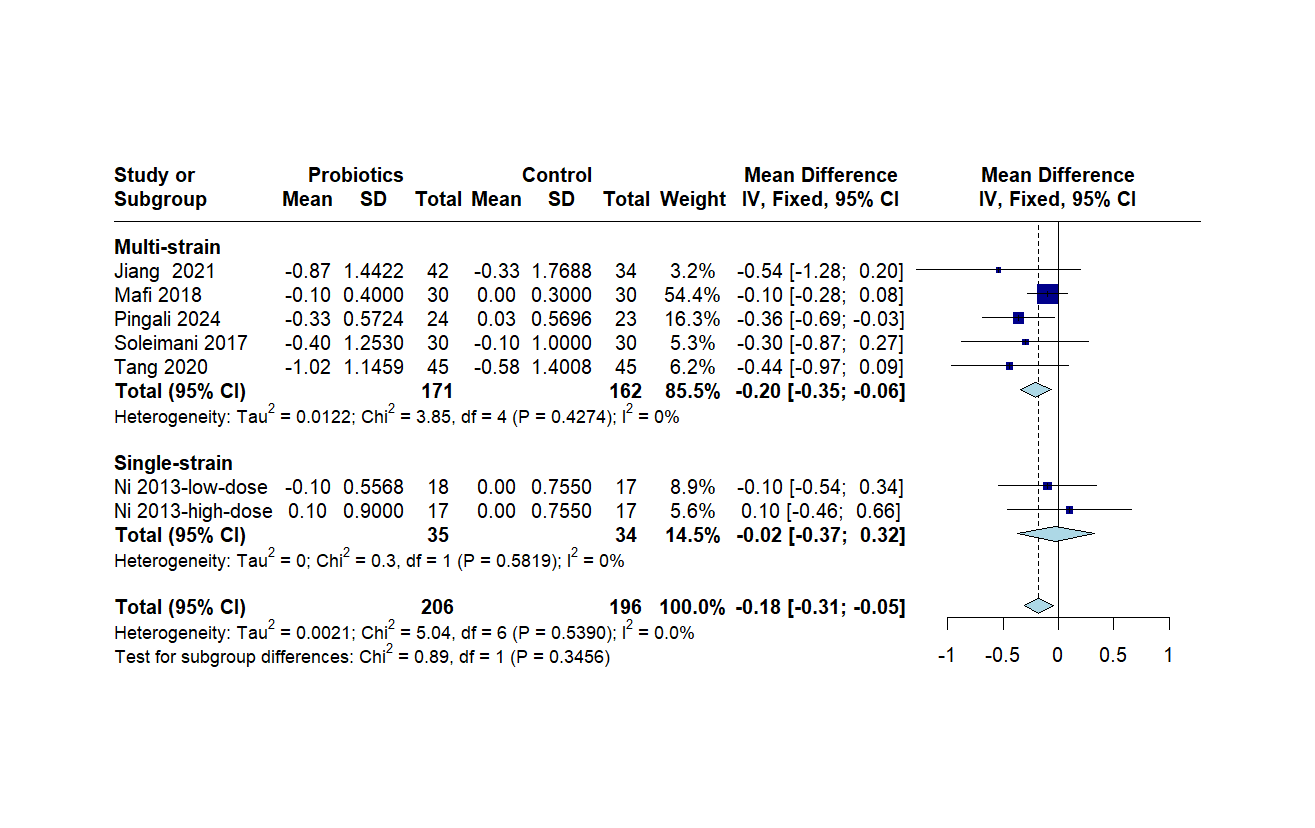


Supplementary Figure 2F. Forest plot depicting mean differences (MD) and the 95% confidence interval (CI) for the impact of probiotic supplementation on lipid profile: triglycerides (TG) levels according to strain composition (single-strain or multi-strain).


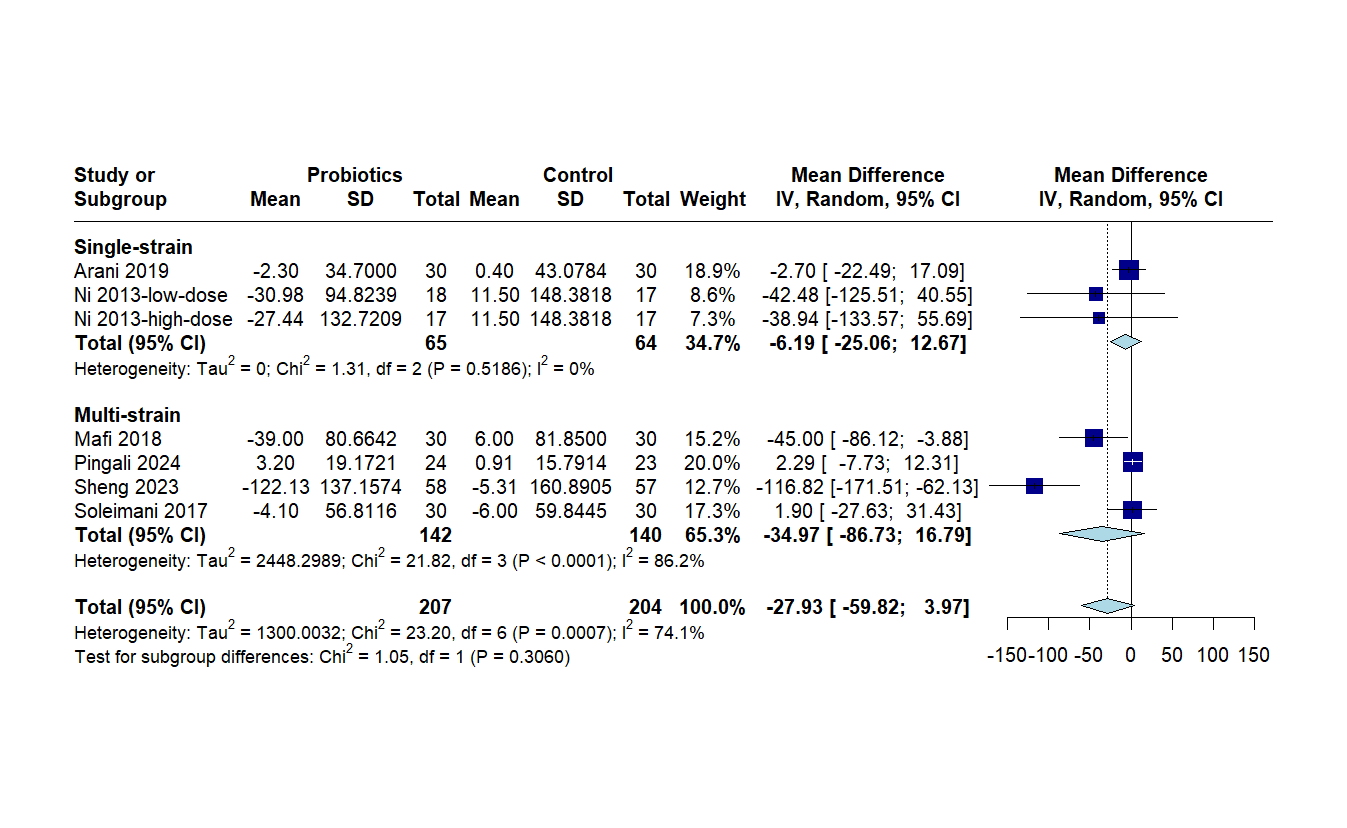


Supplementary Figure 2G. Forest plot depicting mean differences (MD) and the 95% confidence interval (CI) for the impact of probiotic supplementation on total cholesterol (TC) levels according to strain composition (single-strain or multi-strain).


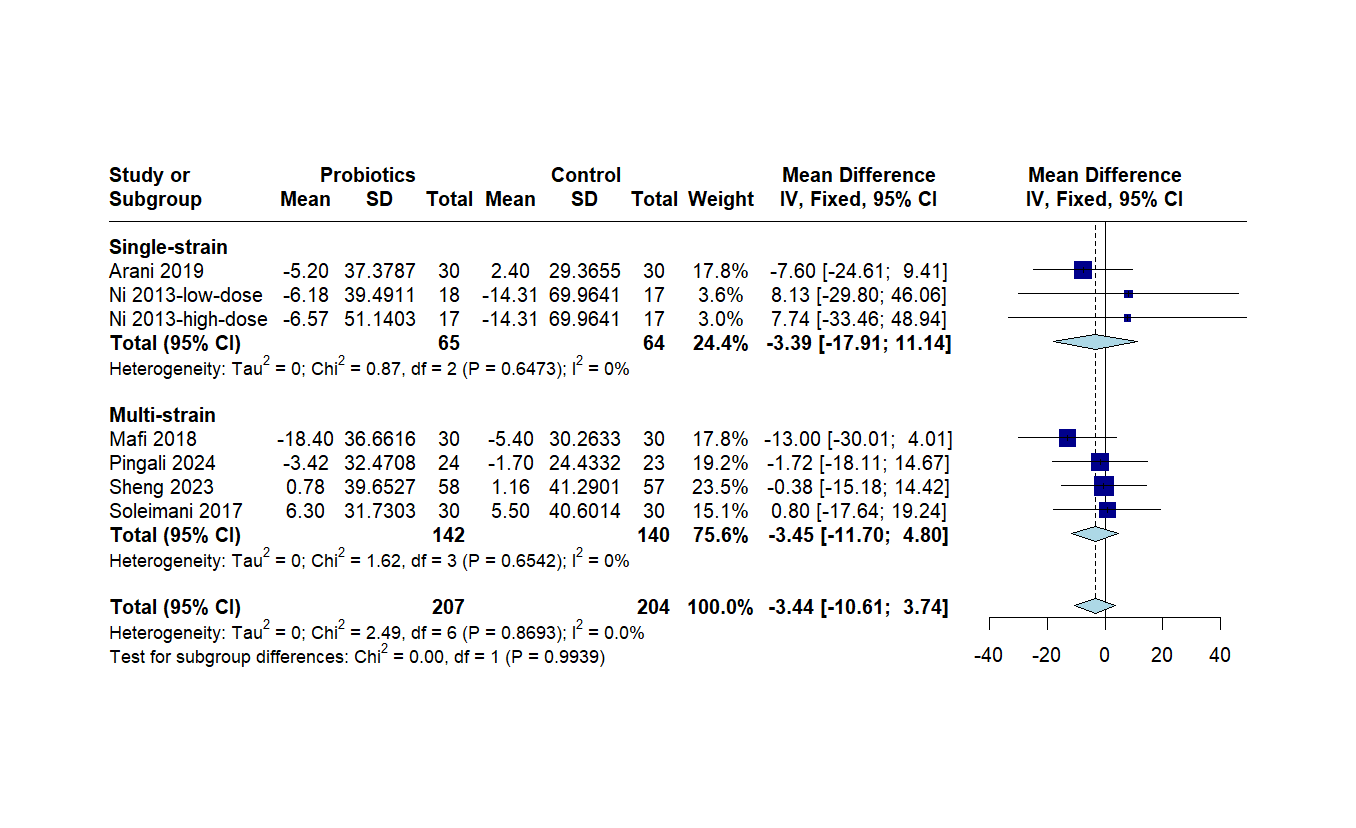


Supplementary Figure 2H. Forest plot depicting mean differences (MD) and the 95% confidence interval (CI) for the impact of probiotic supplementation on high-density lipoprotein cholesterol (HDL-C) levels according to strain composition (single-strain or multi-strain).


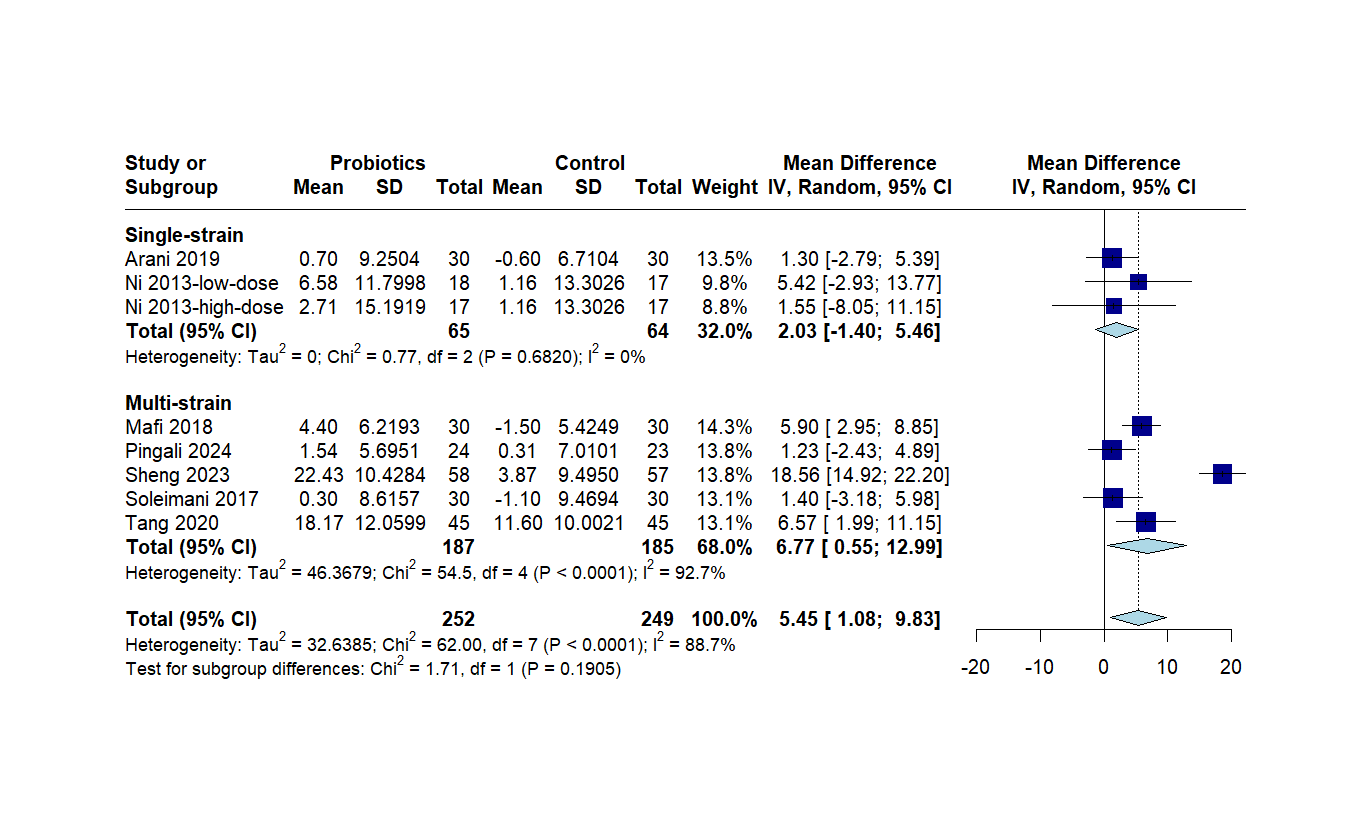


Supplementary Figure 2I. Forest plot depicting mean differences (MD) and the 95% confidence interval (CI) for the impact of probiotic supplementation on low-density lipoprotein cholesterol (LDL-C) levels according to strain composition (single-strain or multi-strain).


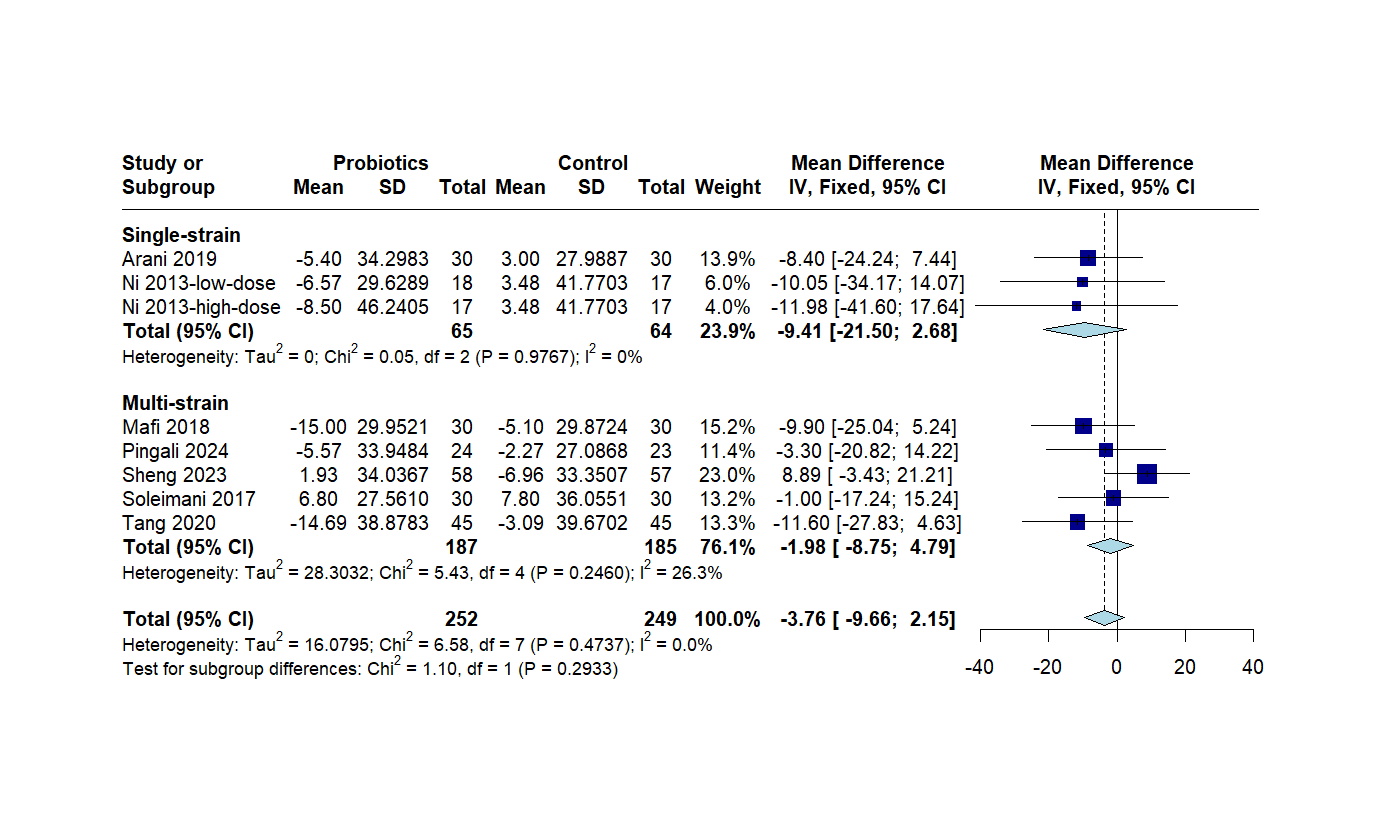

Supplement: Supplementary file 2 [file Table_2.DOCX]
