# Supplementary material for: Effects of probiotic supplementation on diabetic kidney disease: a systematic review and meta-analysis of randomized controlled trials
Source: Front Microbiol. 2026 May 20;17:1760954. doi: 10.3389/fmicb.2026.1760954 (PMC13230064; doi:10.3389/fmicb.2026.1760954)

Supplementary Figure 1. Leave-one-out sensitivity analysis assessing the robustness of the effect of probiotic supplementation on estimated glomerular filtration rate (eGFR) in the 12-week intervention subgroup.


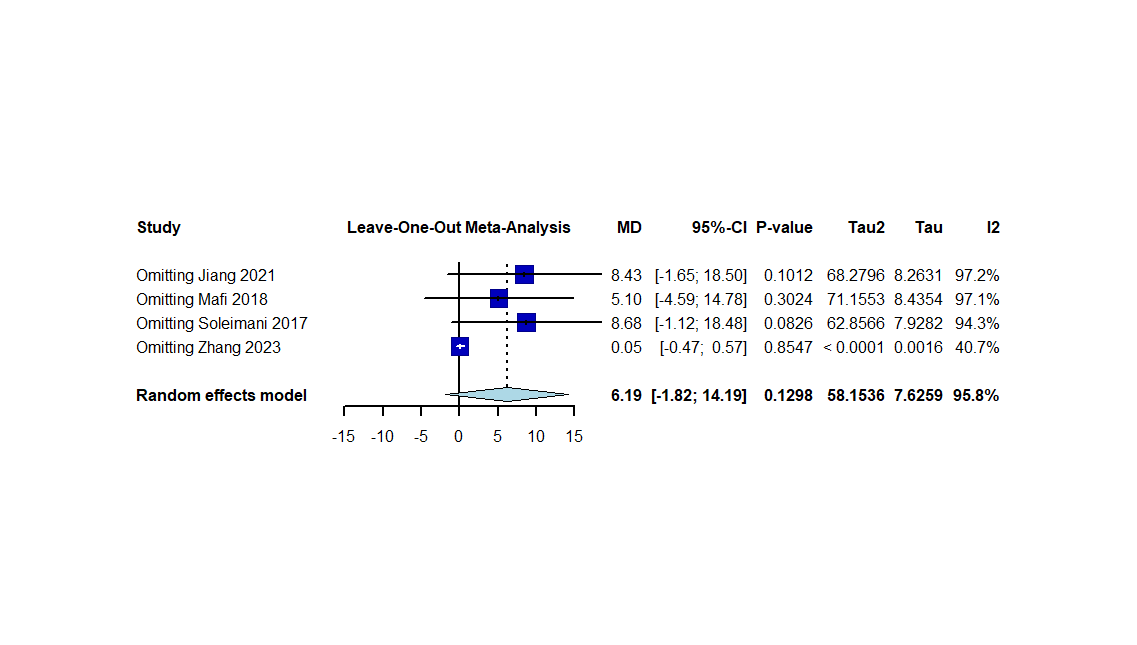


Supplementary Figure 2. Leave-one-out sensitivity analysis assessing the robustness of the effect of probiotic supplementation on blood urea nitrogen (BUN) in the Multi-strain intervention subgroup.


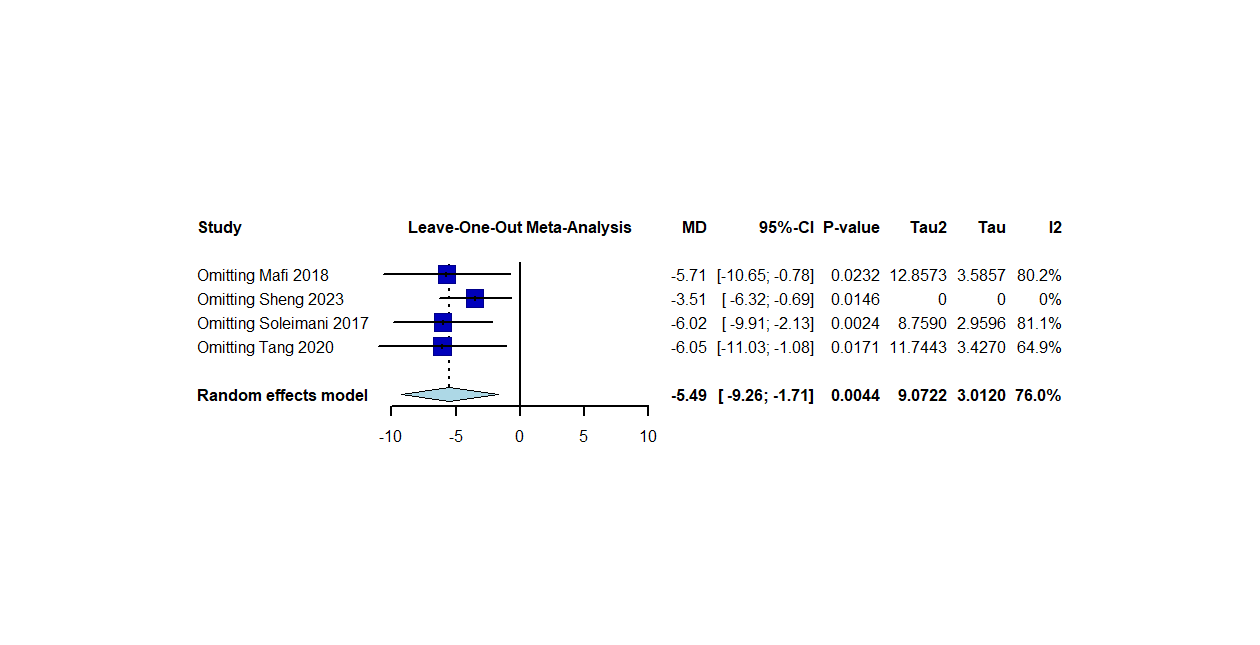


Supplementary Figure 3. A leave-one-out sensitivity analysis of the impact of probiotic supplementation urinary albumin-to-creatinine ratio (UACR).


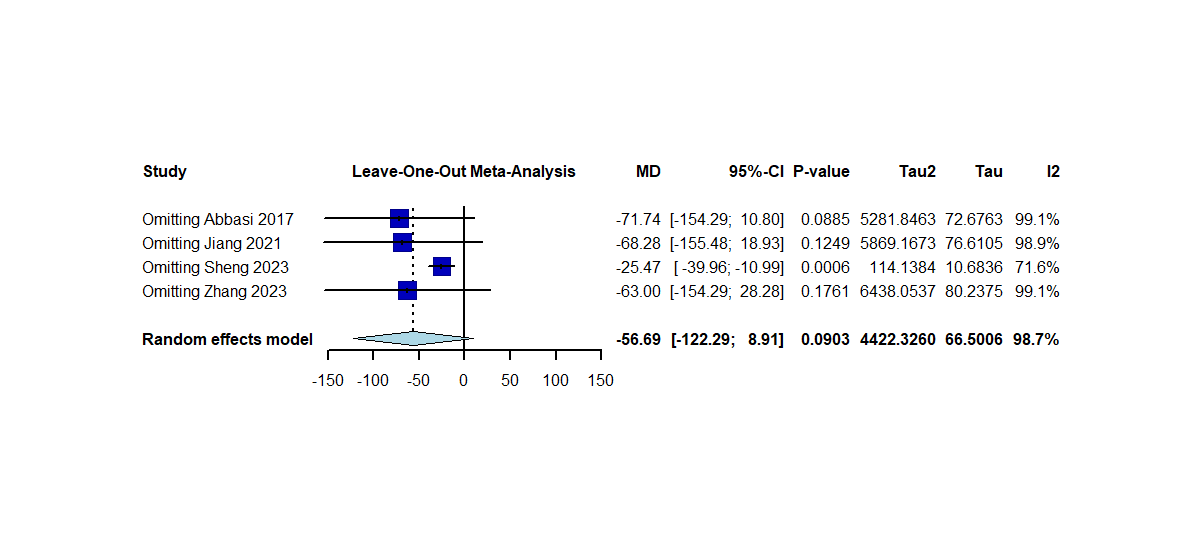


Supplementary Figure 4. A leave-one-out sensitivity analysis of the impact of probiotic supplementation on cystatin C (Cys-C).


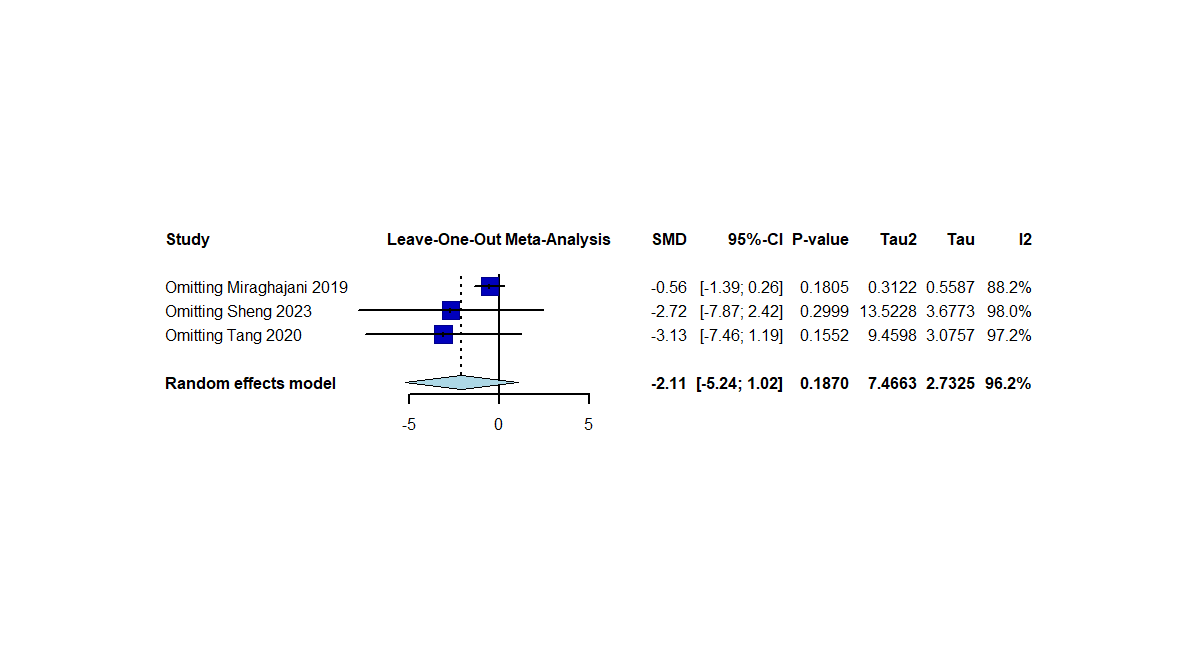


Supplementary Figure 5. Leave-one-out sensitivity analysis assessing the robustness of the effect of probiotic supplementation on fasting plasma glucose (FPG) in the Multi-strain intervention subgroup.


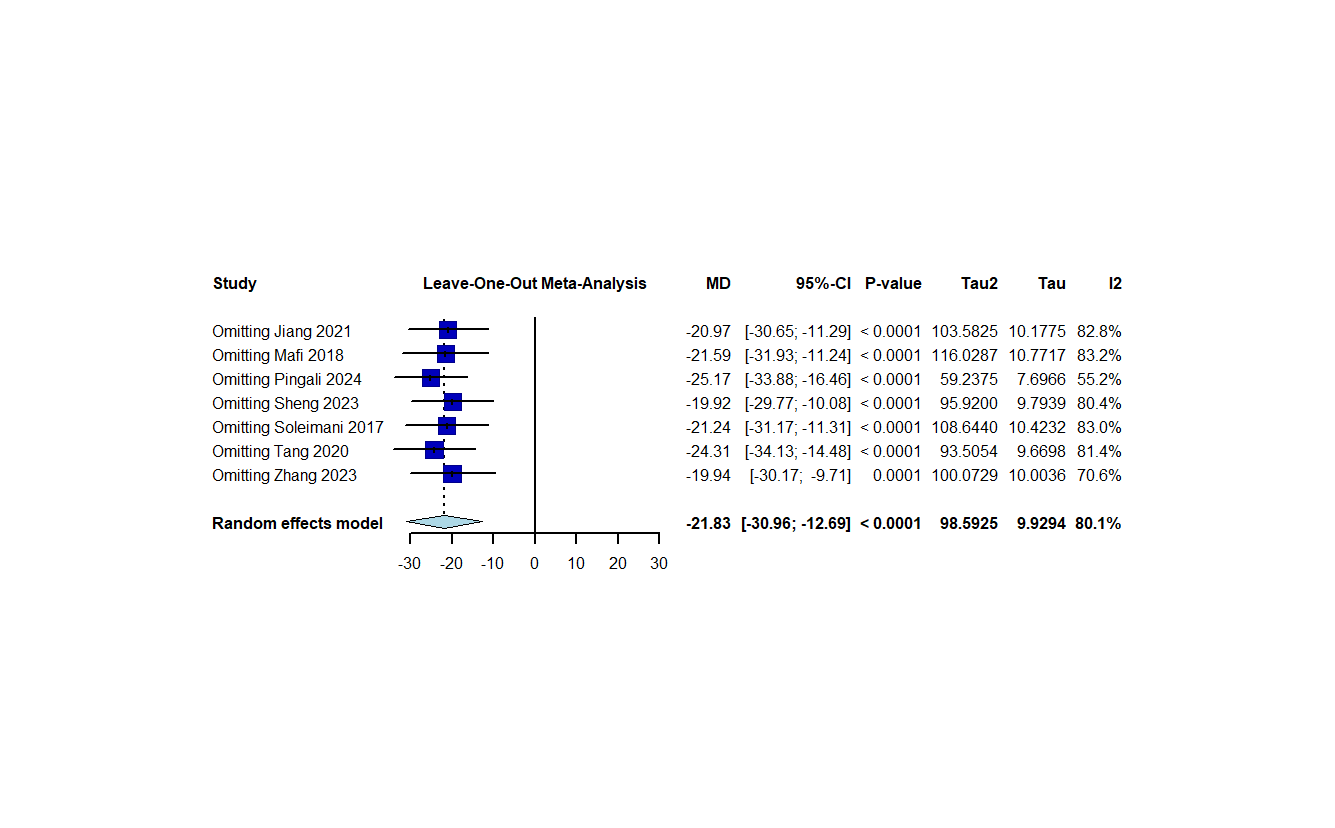


Supplementary figure 6. A leave-one-out sensitivity analysis of the impact of probiotic supplementation on homeostasis model assessment of insulin resistance (HOMA-IR).


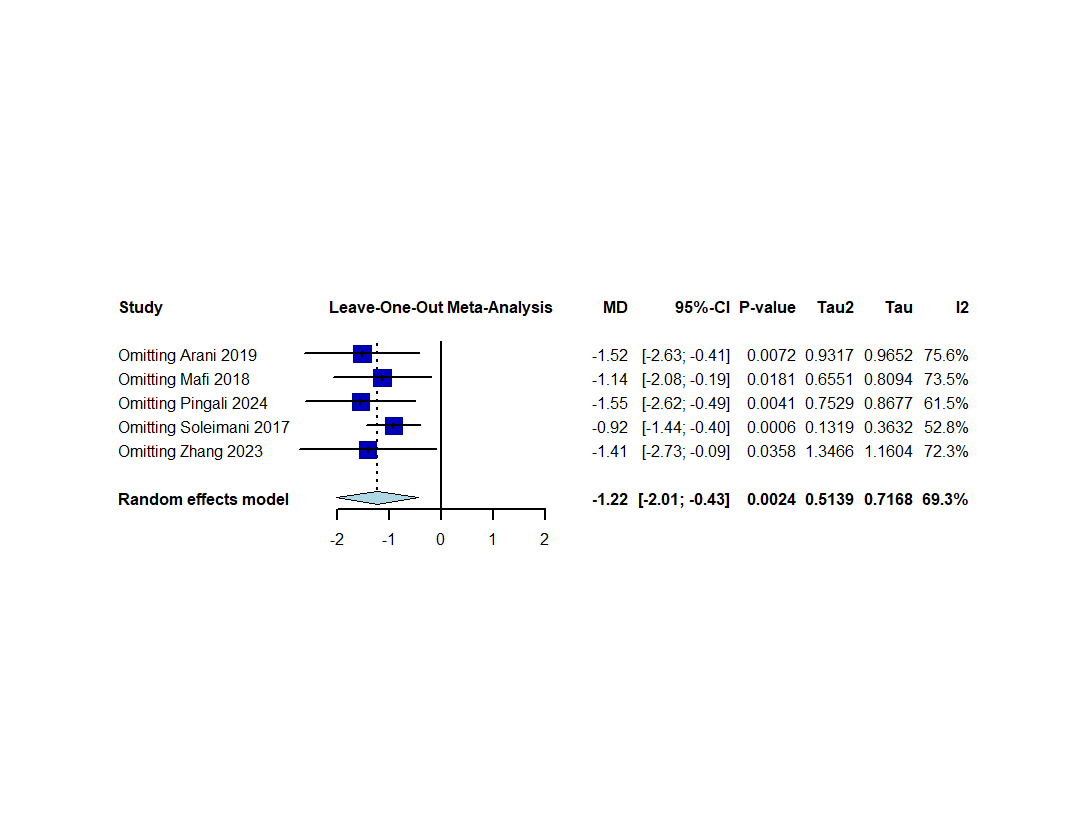


Supplementary Figure 7. Leave-one-out sensitivity analysis assessing the robustness of the effect of probiotic supplementation on triglycerides (TG) in the Multi-strain intervention subgroup.


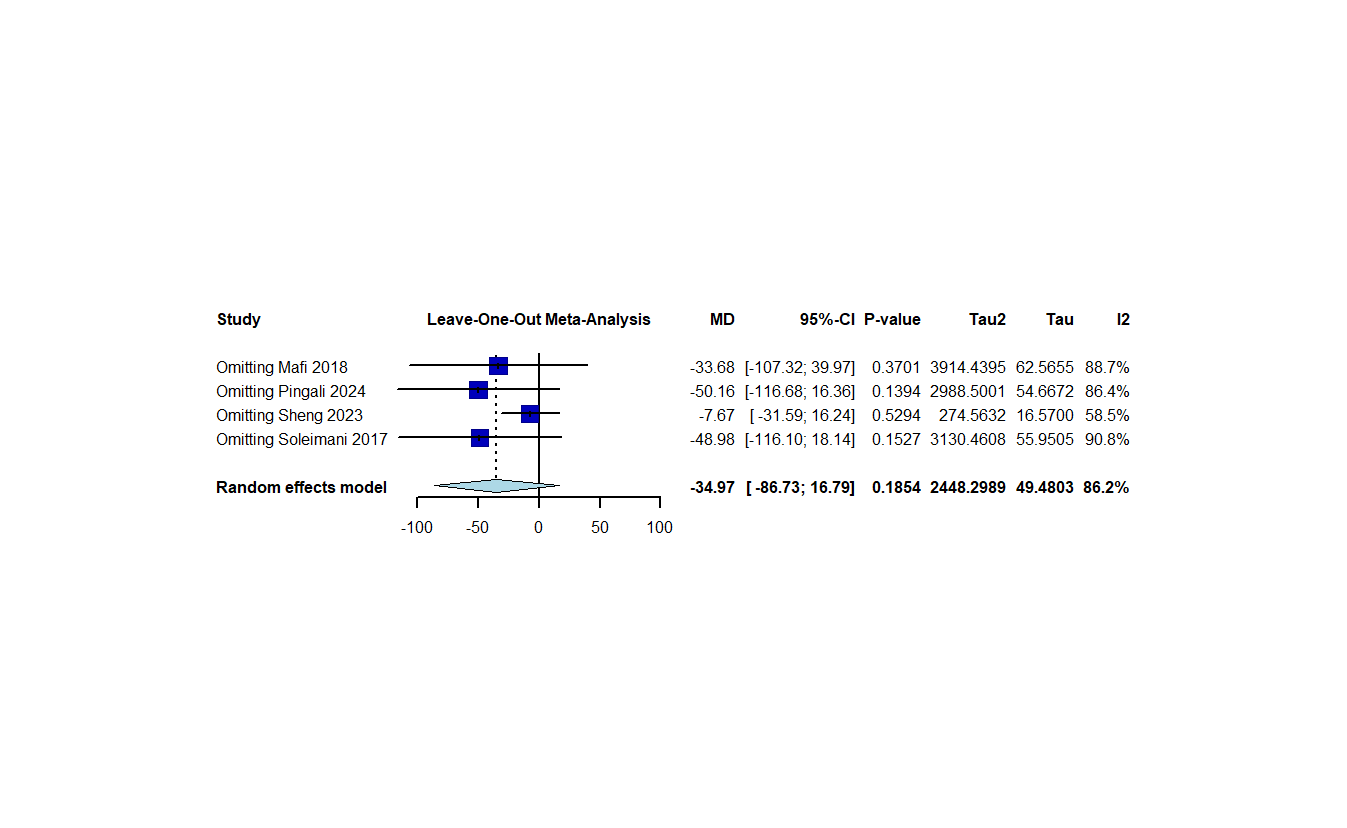


Supplementary Figure 8. Leave-one-out sensitivity analysis assessing the robustness of the effect of probiotic supplementation on high-density lipoprotein cholesterol (HDL-C) in the Multi-strain intervention subgroup.


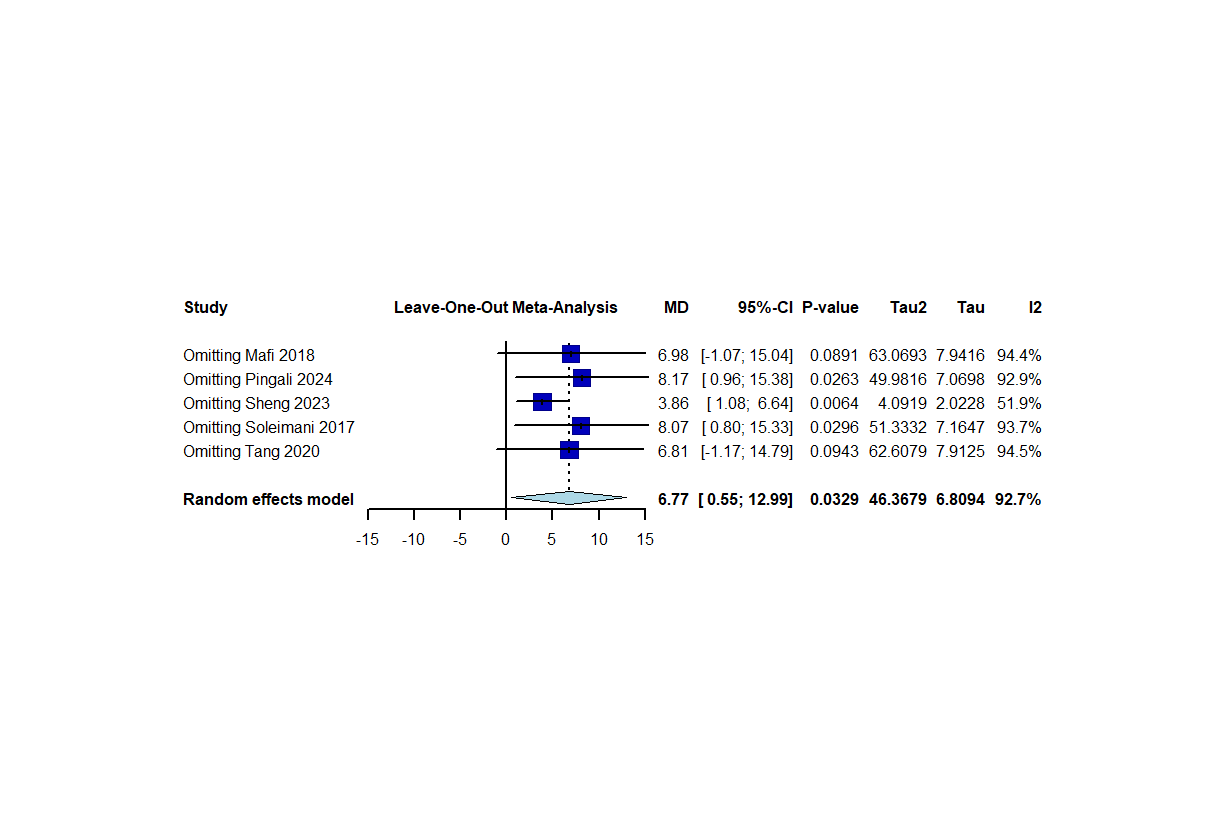


Supplementary Figure 9. A leave-one-out sensitivity analysis of the impact of probiotic supplementation on nitric oxide (NO).


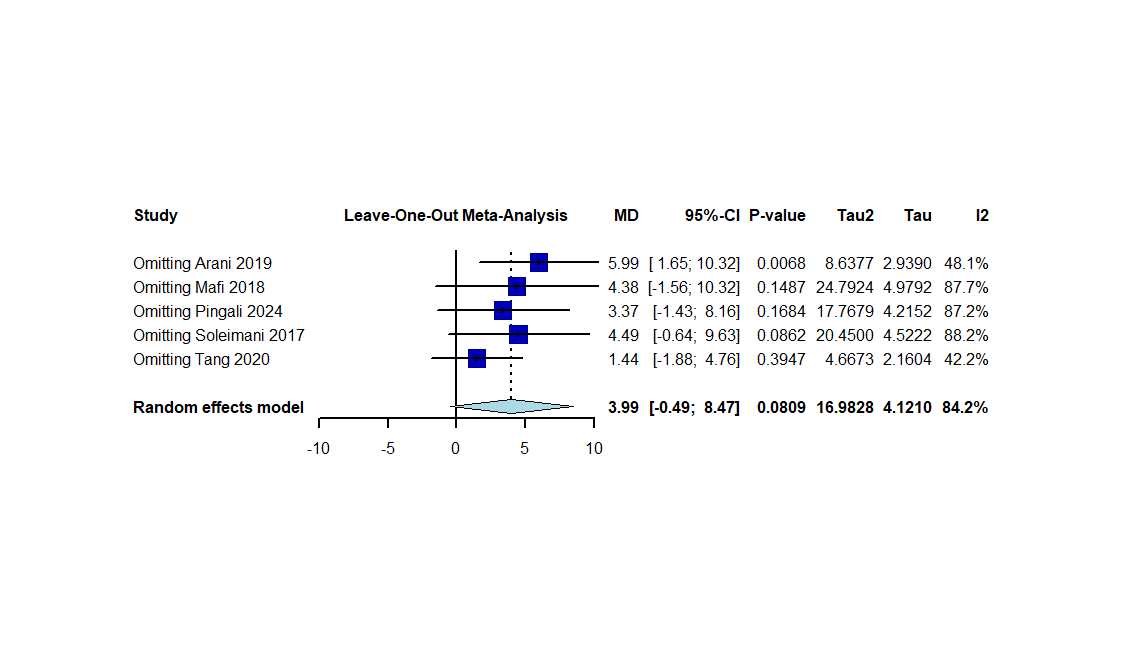

Supplement: Supplementary file 3 [file Table_3.DOCX]
