# Supplementary figures and images for: Effects of probiotic supplementation on diabetic kidney disease: a systematic review and meta-analysis of randomized controlled trials
Source: Front Microbiol. 2026 May 20;17:1760954. doi: 10.3389/fmicb.2026.1760954 (PMC13230064; doi:10.3389/fmicb.2026.1760954)

Supplementary Figure 1. Fasting plasma glucose (FPG) funnel plot.


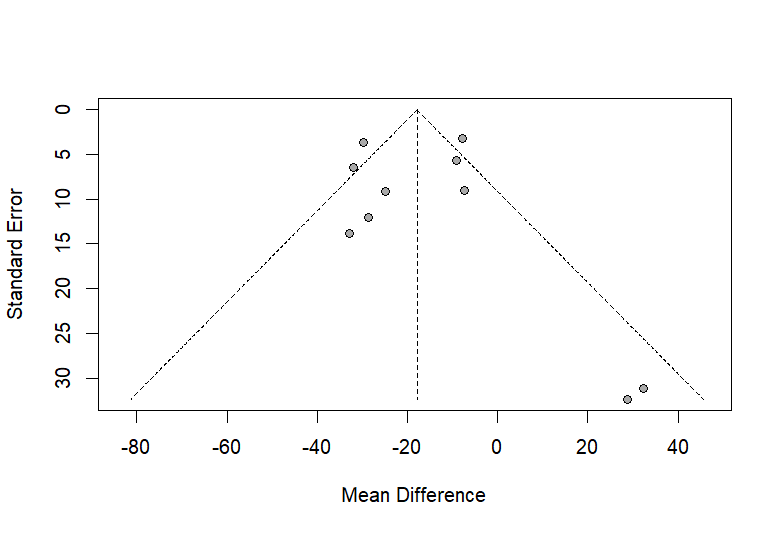

Supplement: Supplementary file 4 [file Table_4.DOCX]
